# Tumour priming by ultrasound mechanogenetics for CAR T therapy

**DOI:** 10.1038/s41563-025-02391-8

**Published:** 2025-10-31

**Authors:** Chi Woo Yoon, Chunyang Song, Dung Ngo Minh Nguyen, Xi Yu, Linshan Zhu, Phuong Ho, Ziliang Huang, Gengxi Lu, Yuxuan Wang, Fan Wei, Yunjia Qu, Ali Zamat, Alexa Lewis, Ruimin Chen, Yushun Zeng, Priyankan Datta, Nan Sook Lee, Christina Jamieson, Bingfei Yu, K. Kirk Shung, Qifa Zhou, Longwei Liu, Yingxiao Wang

**Affiliations:** 1 The Shu Chien-Gene Lay Department of Bioengineering and Institute of Engineering in Medicine, University of California, San Diego, La Jolla, CA USA; 2 The Alfred E. Mann Department of Biomedical Engineering, Viterbi School of Engineering, University of Southern California, Los Angeles, CA USA; 3 Roski Eye Institute, Department of Ophthalmology, Keck School of Medicine, University of Southern California, Los Angeles, CA USA; 4 Department of Aerospace and Mechanical Engineering, University of Southern California, Los Angeles, CA USA; 5 Department of Urology and Moores Cancer Center, University of California, San Diego, La Jolla, CA USA; 6 Department of Molecular Microbiology and Immunology, Keck School of Medicine, University of Southern California, Los Angeles, CA USA

**Keywords:** Synthetic biology, Cancer immunotherapy

## Abstract

Cell-based cancer immunotherapy holds potential as a therapeutic approach, yet its application for solid tumour treatment remains challenging. Here we report a focused-ultrasound-based approach that mechanically induces the localized expression of CD19 antigen within a subpopulation of cells within solid tumours, which function as local ‘training centres’ to activate chimeric antigen receptor T cells. Activated chimeric antigen receptor T cells attack the whole cancer cell population near the tumour site, thus achieving cancer suppression. The system achieves targeted gene expression by integrating focused-ultrasound-triggered mechanical stimulation and the subsequent calcium response of cancer cells with a doxycycline-gated AND-logic genetic circuit, both of which need to be active for effective induction of CD19 expression. We validate the functionality of the approach in vitro, in organoids and in vivo, achieving direct control of user-designed gene expressions through FUS-mediated mechanical stimulation without the need of any cofactor, demonstrating the approach’s potential as a versatile platform for precisely controllable immunotherapy. Overall, our combinatorial approach offers a focused-ultrasound-controlled remote and non-invasive priming of solid tumours for effective and safe chimeric antigen receptor T cell immunotherapy via the induced production of clinically validated antigens.

## Abstract (web summary)

A focused-ultrasound-mediated mechanogenetics approach enables the genetic modification of cancer cells near solid tumours to activate chimeric antigen receptor T cell response and achieve tumour suppression at distinct sites.

## Main

The ability to remotely control genetics and cellular functions with high spatiotemporal precision has long been a goal in the field of biomedicine. Ultrasound is a promising external modulator for regulating cellular processes, as it is a form of mechanical energy that can be transmitted efficiently through deep tissues, offering a penetration depth that is several orders higher than that of light^1,2^. Previously, ultrasound has been recognized for its ability to activate genes via thermo effects^3–5^, which are a by-product of ultrasound energy being converted into heat due to the frictional forces and viscous damping within the tissues upon absorption^6^. However, recent advancements in our understanding of mechanotransduction pathways in cells^7^ has broadened our perspective on ultrasound’s potential. These developments may allow an additional modality for remote control of genetics and cellular behaviours with a higher precision in space and time^8–10^. We previously developed mechanogenetics, in which focused ultrasound (FUS) is used to activate specific mechanosensitive channels, called Piezo1, in cells physically coupled to microbubbles^11^. However, the in vivo application of this approach is limited by the uncontrollability of this microbubble co-factor, together with its large size^12^ and relatively short circulation time in the body^13^. Thus, an unmet need remains for FUS-based mechanogenetics that can achieve specific genetic and cellular control directly via mechanical perturbation without the need of co-factors such as microbubbles.

Chimeric antigen receptor (CAR) T cell therapy is a groundbreaking immunotherapy that involves the genetic modification of a patient’s T cells to express CARs, which can specifically target and eliminate cancer cells^14^. This approach has shown remarkable success in treating haematological malignancies, revolutionizing cancer therapy. However, the application of CAR T cell (CAR T) therapy in solid tumours faces major obstacles, including the challenge posed by the heterogeneity of solid tumours, which complicates the discovery of truly tumour-specific and homogeneous antigens and makes it difficult to avoid life-threatening on-target off-tumour toxicity^15^. The remote and non-invasive control of genetics and cell activity with a high spatiotemporal precision would be a potential solution to precisely address this issue, providing a more spatiotemporally targeted and adaptable approach for solid tumour treatment.

In this study we present a FUS-based mechanogenetics approach that enables remote and non-invasive control over designed cellular genetics tailored to leverage the mechanosensitivity of cancer cells in calcium signalling, paired with a doxycycline-gated AND-logic genetic circuit (both of the two inputs need to be turned on to activate the output). This system has been engineered to induce the production of a clinically validated antigen within a subpopulation of cancer cells, creating local ‘training centres’ that could prime and guide synthetic Notch (synNotch) CAR T cells to effectively control and suppress the cancer population across the tumour regions through recognition of a homologous antigen. As such, our technology provides potential for precise FUS-controlled tumour targeting using clinically validated antigens, aiming to improve the safety and efficacy of CAR T immunotherapy.

## FUS induces calcium signalling in PC-3 cancer cells

**Fig. 1:**
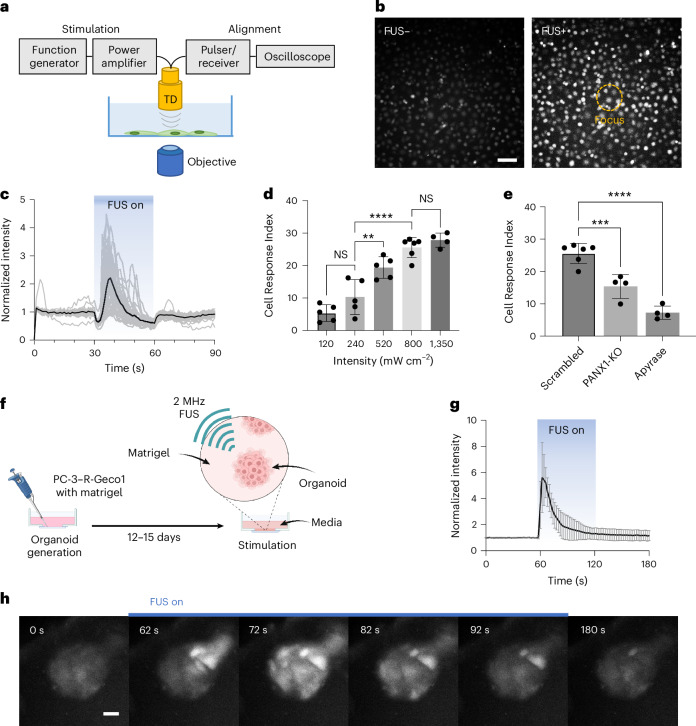
FUS-induced calcium responses in prostate cancer PC-3 cells. **a**, Schematic of the FUS stimulation apparatus integrated with an inverted epifluorescence microscope to monitor calcium dynamics in real time (TD, transducer). **b**, Representative images of intracellular calcium responses in Fluo-4 AM-loaded PC-3 cells before (FUS−) and after FUS (FUS+). Orange dashed circle, FUS focal zone (120 µm). Scale bar, 100 µm. **c**, Time courses of intracellular calcium in individual PC-3 cells (grey lines, *n* = 47) within the focal zone; black line, mean trace. **d**, Quantification of calcium responses at 2 MHz FUS with varying intensities (spatial-peak, temporal-average intensity, *I*_SPTA_) of 120 (*n* = 5), 240 (*n* = 5), 520 (*n* = 5), 800 (*n* = 6) and 1,350 mW cm^−2^ (*n* = 4) using the Cell Response Index. Adjusted *P* values are as follows: 240 versus 520, *P* = 0.0051; 240 versus 800, *P* < 0.0001. **e**, Effects of *PANX1* knock-out (KO; *n* = 4) and apyrase (*n* = 4) on FUS-induced calcium responses compared with scrambled control (*n* = 6). Adjusted *P* values are as follows: scrambled versus *PANX1*-KO, *P* = 0.0006; scrambled versus apyrase, *P* < 0.0001. **f**, Schematic of PC-3-derived organoid culture set-up. **g**, Calcium response profiles of PC-3-derived organoids (*n* = 5) expressing R-Geco1 upon FUS stimulation (60–120 s). **h**, Representative time-lapse images of FUS-induced calcium responses in PC-3-derived organoids. Scale bar, 50 µm. Data in **d**, **e** and **g** are shown as mean ± s.d. Statistical analyses were performed using one-way analysis of variance (ANOVA) with Tukey’s (**d**) or Dunnett’s (**e**) multiple comparisons tests (two-sided). A biological replicate is defined as an independent dish or organoid culture. Images in **b** and **h** are representative of ≥5 independent experiments. **, *** and **** indicate adjusted *P* < 0.01, *P* < 0.001 and *P* < 0.0001, respectively. Panel **f** created with BioRender.com. Source data

Our first goal was to identify a cancer cell that is sensitive to mechanical perturbation by short pulsed acoustic waves without microbubbles or any other co-factor, and that exhibits robust intracellular calcium responses. We selected prostate cancer PC-3 cells for our study, as their sensitivity in calcium signalling to FUS stimulation has been reported previously^16,17^. To assess the FUS-mediated intracellular calcium response, we integrated an ultrasound stimulation system with an epifluorescence microscope (Fig. 1a). For the stimulation, we first employed a single-element 35 MHz FUS transducer with parameters (Methods) that are consistent with previous reported conditions^16,17^, aiming to elicit a strong calcium response that could subsequently drive strong gene expressions while avoiding cell toxicity^18^. This transducer, operating within the low-intensity pulsed ultrasound (LIPU) range, delivers focused energy to a localized region measuring 120 µm in diameter (Supplementary Fig. 1a), allowing for precise mechanistic studies in the culture dish.

We observed strong calcium reactions in response to FUS stimulation (Fig. 1b,c). Interestingly, the cells located outside the focal area of the FUS also showed responses as a form of calcium propagation (Supplementary Fig. 2a). We also observed a response latency of several seconds from the onset of the FUS stimulation (Fig. 1c). This response latency and the calcium propagation beyond the FUS-stimulated area can be attributed to the paracrine release of ATP by the mechanosensitive non-junctional hemichannels, as previously reported^16^. These hemichannels, known for their sensitivity to mechanical stimuli, have been shown to release ATP into the extracellular space upon activation^19–22^. The extracellular ATPs trigger the activation of purinergic receptors, leading to both calcium influx and intracellular calcium release in neighbouring cells. As such, while FUS can directly stimulate ATP release through mechanical sensing, the overall calcium response is an indirect effect, mediated by the subsequent purinergic signalling^16^. This highlights the intricate interplay between mechanical and biochemical signalling pathways in FUS-induced cellular responses. Indeed, we detected a significantly increased level of extracellular ATP from the dish with FUS exposure^16^ (Supplementary Fig. 2b).

We further investigated these FUS responses using a three-dimensional (3D) culture model to better mimic the physiologically relevant conditions. We employed a clinically relevant low-frequency 2 MHz FUS transducer, as the use of high-frequency FUS hinders clinical adaptation due to it shallow penetration. To optimize the output power of the 2 MHz FUS for inducing robust calcium responses, we first calibrated various FUS intensities in two-dimensional (2D) cultures (120, 240, 520, 800 and 1,350 mW cm^−2^ spatial-peak temporal-average intensity) within the LIPU range (Fig. 1d). The results showed a clear increase in calcium responses with higher intensities, with saturation observed in the 520–800 mW cm^−2^ range. To maintain the acoustic power exerted on the targets within a similar range as that in the 2D studies, we selected 800 mW cm^−2^ for further 3D experiments (Supplementary Fig. 1b).

To validate whether the hemichannels play a pivotal role in the mechanosensing of low-frequency FUS, we first knocked out pannexin1 (*PANX1*), a well-established mechanosensitive hemichannel, using the CRISPR ribonucleoprotein (RNP) technique, and monitored the FUS-induced responses. The results showed that FUS-mediated calcium responses were significantly reduced in *PANX1* knock-out cells (Fig. 1e), indicating that hemichannels are involved in this process. We further treated the cells with apyrase, which degrades extracellular ATP. Apyrase treatment led to a complete reduction in calcium responses, suggesting that the calcium responses are dominantly dependent on FUS-induced ATP release (Fig. 1e). These results demonstrate that the 2 MHz FUS-induced calcium responses are mediated by an ATP-release-dependent mechanism, consistent with the pathways reported for 35 MHz FUS.

We then generated a 3D organoid model based on PC-3 cells that stably express the genetically encoded calcium sensor R-Geco1 (Fig. 1f), following the previously reported methods for organoid generation^23^. The sizes of the matured organoids ranged from 200 to 600 µm, which aligns well with the focal zone of the 2 MHz FUS (~700 µm; Supplementary Fig. 1b). We observed that PC-3 cells in the 3D organoids responded to FUS with strong calcium signals (Fig. 1g,h). These calcium waves spread from the stimulated area to neighbouring cells, reinforcing the involvement of paracrine signalling. These findings suggest that FUS-induced mechanical perturbation is sufficient to induce intracellular calcium responses in both 2D and 3D culture models.

## FUS-induced calcium signalling is conserved across cancers

To determine whether the mechanotransduction effect of FUS, observed as a calcium influx in PC-3 cells, is a general feature across different cell types, we extended our investigation to include MDA-MB-231, a human breast cancer cell line, and U-87MG, a human glioblastoma cell line. To establish a 3D culture model adaptable to various cancer cells, we employed a magnetic-field-assisted assembly technique^24^ to generate spheroids and applied FUS. Both cancer cell lines were sensitive to mechanical perturbations induced by FUS, displaying robust calcium responses similar to those observed in PC-3 cells (Supplementary Fig. 3a,b). This result underscores the potential of FUS as a general tool for triggering intracellular signalling across different cell types.

To confirm that these calcium responses were not a secondary effect of mechanical or thermally induced cell damage, we assessed spheroid viability following FUS stimulation. Live/dead staining of U-87MG spheroids showed no significant increase in cell death 72 h after FUS treatment, whereas the apoptotic reagent paclitaxel (200 nM) induced clear cytotoxicity (Supplementary Fig. 4a,b). Additionally, temperature measurement confirmed that FUS exposure did not induce a significant temperature increase in spheroids (Supplementary Fig. 4c). Together, these results demonstrate that FUS-driven mechanotransduction can activate intracellular calcium signalling without compromising cell viability or inducing thermal damage, further supporting its utility as a precise and non-destructive approach for cellular modulation.

## Design of an inducible AND-gated gene circuit

**Fig. 2:**
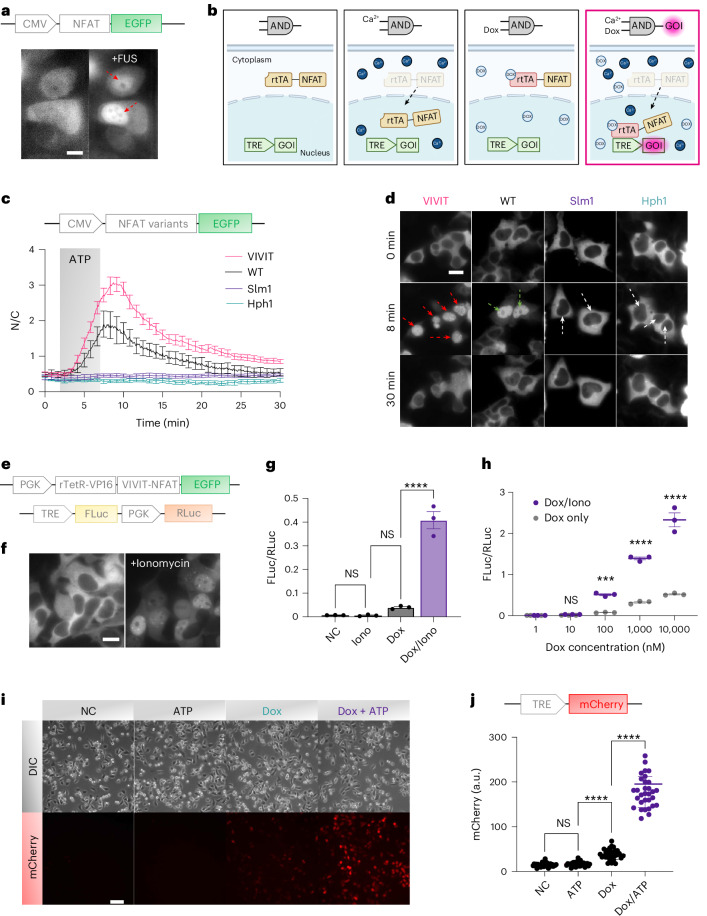
Development and characterization of the CaDox system. **a**, NFAT nuclear translocation following repeated FUS stimulations, visualized via EGFP-tagged NFAT. Scale bar, 10 µm. **b**, Schematic of the calcium-gated and doxycycline-gated genetic circuit (CaDox). CMV, cytomegalovirus promoter; GOI, gene of interest. **c**,**d**, Time courses (**c**) and representative images (**d**) of NFAT translocation with variants exhibiting different calcineurin affinities. Red and green arrows in **a** and **d** indicate nuclei showing NFAT translocation, whereas white arrows in **d** mark nuclei without detectable translocation. ATP (60 µM) was applied at 2 min and washed off at 7 min (*n* = 5 biological replicates). N/C, nuclear over cytoplasmic ratio; WT, wild type. **e**, Diagram of the CaDox circuit comprising the CaDox regulator and reporter modules. PGK, phosphoglycerate kinase promoter. **f**, CaDox regulator translocation in response to ionomycin (1 µM). **g**, Quantification of CaDox-mediated gene induction using FLuc as the inducible reporter and RLuc as the constitutive control (*n* = 3 per group). Statistical significance was assessed using one-way ANOVA followed by Tukey’s multiple comparisons test (two-sided). Adjusted *P* value is as follows: Dox versus Dox/Iono, *P* < 0.0001; Dox, doxycycline. Iono, ionomycin; NC, negative control. **h**, Dose–response curve of doxycycline concentration in the presence or absence of ionomycin (*n* = 3 per group). Statistical significance was assessed using two-way ANOVA with Šidák’s multiple comparisons test (two-sided). Adjusted *P* values are as follows: 100 nM, *P* = 0.0001; 1 µM, *P* < 0.0001; 10 µM, *P* < 0.0001. **i**,**j**, Representative images (**i**) and quantifications (**j**) of mCherry reporter expression in PC-3 cells under indicated conditions (*n* = 31). DIC, differential interference contrast. One-way ANOVA with Tukey’s multiple comparisons test (two-sided); adjusted *P* values are as follows: ATP versus Dox, *P* < 0.0001; Dox versus Dox/ATP, *P* < 0.0001. Scale bar, 100 µm. Data in **g**, **h** and **j** are represented as mean ± s.e.m. Biological replicates were defined as independent wells or dishes. Images in **a**, **d**, **f** and **i** are representative of ≥3 independent experiments. *** and **** denote adjusted *P* < 0.001 and *P* < 0.0001, respectively. NS, not significant. Panel **b** created with BioRender.com. Source data

We then explored the possibility of designing genetic transducers and circuits that can convert the accumulated effect of FUS-induced calcium dynamics into controlled gene expressions. Nuclear factor of activated T cells (NFAT) is a family of transcription factors that play important roles in regulating gene expression in immune cells as well as in other cell types, including cancer cells^25^. NFAT proteins can be activated by intracellular calcium signalling and subsequently translocate to the nucleus to regulate the transcription of target genes. To investigate whether the FUS-mediated calcium responses in PC-3 cells can be sufficient to induce NFAT translocation, we introduced a lentiviral vector to express NFAT protein fused with enhanced green fluorescent protein (EGFP) in PC-3 cells. A clear translocation of NFAT proteins was observed upon repetitive FUS stimulations (Fig. 2a and Supplementary Fig. 2c), suggesting that the NFAT translocation may be used to control transcriptional activity on FUS-mediated calcium.

Next we aimed to develop a genetic transducing module that can transduce FUS-induced calcium signalling into user-defined gene expressions. We previously developed a genetic circuit coupling a minimal promoter with the NFAT response element (NFAT-RE) and two other calcium-dependent elements—the cAMP response element (CRE) and the serum response element (SRE)—to transduce calcium signals into gene expressions^11^. However, besides the varying expression levels of endogenous NFAT in different individual cells^26^, this system can be vulnerable to spontaneous calcium noises in cancer cells^27^. We indeed found that a genetic transducer based on NFAT-RE had a strong background noise of genetic activities in PC-3 cells, reaching levels comparable to the calcium ionophore ionomycin-induced gene production monitored by a GFP reporter (Supplementary Fig. 5).

To mitigate the noisy calcium-mediated genetic background as well as the varying levels of endogenous NFAT activity in individual PC-3 cells, we decided to engineer a synthetic transcription activator (STA) based on reverse tetracycline transactivator (rtTA) and NFAT protein. The rtTA can regulate gene expression by binding to the tetracycline operator (tetO) DNA sequence in the presence of the antibiotic tetracycline or its derivative doxycycline^28^. Thus, by directly fusing the rtTA with the NFAT, this STA functions as an AND-logic circuit, requiring the presence of both calcium signalling for rtTA–NFAT translocation and doxycycline for STA–DNA interaction to initiate transcription (Fig. 2b).

We first improved the STA by engineering NFAT with enhanced shuttling efficiency by modifying its calcineurin docking sites. Calcineurin is a calcium-dependent phosphatase that becomes activated when calcium levels increase, leading to NFAT dephosphorylation and translocation to the nucleus^29^. Thus, we hypothesized that by modifying the calcineurin binding sites in NFAT, we might alter its translocation efficiency. We selected several mutants of the calcineurin docking sequence with different dissociation constants (*K*_d_; Supplementary Fig. 6a) and used them to engineer GFP-tagged NFAT variants to track their translocation upon ATP-induced calcium dynamics (Supplementary Fig. 6b). The imaging results showed that the binding affinities between NFAT and calcineurin can substantially affect the translocation of NFAT. The VIVIT (Val–Ile–Val–Ile–Thr) variant, which had the lowest *K*_d_ (0.5 µM), exhibited the fastest and strongest translocation upon calcium increase induced by ATP (Fig. 2c,d). Interestingly, by slightly lowering the binding affinities (*K*_d_ of 25 μM to 40 μM), NFAT completely lost its shuttling capability upon calcium stimulation. This result showed that the engineered NFAT variants with better binding affinities towards calcineurin can enhance the translocation capability, which could potentially improve the efficacy of genetic and transcriptional regulation in response to FUS stimulation.

We further truncated the DNA-binding domain (Rel Homology Domain) of NFAT to avoid potential interference from endogenous NFAT pathways, while retaining the calcium-dependent nuclear translocation capability of NFAT. This truncated NFAT (tNFAT; amino acids 4–399 of NFAT1) can indeed shuttle efficiently between the cytoplasm and nucleus upon calcium signalling (Supplementary Fig. 6c). We then created the STA by fusing the tNFAT containing the high-affinity VIVIT mutant sequence (VIVIT-tNFAT) with rtTA to enable gating by doxycycline. The resulting STA was hence designed to have its gene expression precisely controllable by the doxycycline dosage. We named this calcium-gated and doxycycline-gated STA the CaDox regulator, and its corresponding tetracycline response element (TRE)-based reporter circuit the CaDox reporter. Together, these components compose the CaDox system (Fig. 2e).

We verified the calcium-dependent translocation of the CaDox regulator by tracking EGFP (Fig. 2f), which showed efficient translocation upon calcium elevation induced by 1 µM ionomycin. We then examined whether the CaDox system could be tuned to minimize the transcriptional leakage engendered from spontaneous calcium noise. To conduct gene expression assays, we established a dual luciferase reporter system with inducible firefly luciferase (FLuc) and constitutive renilla luciferase (RLuc) as an internal reference in HEK293T cells. The cells were treated with doxycycline and/or ionomycin for 1 h and then washed before being subjected to a luciferase assay 6 h after the drug treatment. The results showed that the CaDox system had minimal leakage in the absence of doxycycline (Fig. 2g). While minor background noise occurred when the cells were incubated with doxycycline only, significant gene inductions were observed only in the presence of both calcium and doxycycline (Fig. 2g). The level of induction could be precisely controlled by the dosage of doxycycline (Fig. 2h), allowing tunability to meet the varying needs of different applications. Using mCherry as the reporter, the CaDox system also showed a gated induction by doxycycline and ATP-induced calcium in PC-3 cells (Fig. 2i,j). This CaDox system is applicable across different cell types, including the Jurkat T cell line and MDA-MB-231 breast cancer cells (Supplementary Fig. 7). As such, we engineered an AND-logic gene circuit, the CaDox system, which can induce efficient calcium-mediated gene expression gated by doxycycline with minimal background noise.

## FUS activates CaDox for gene control in vitro

**Fig. 3:**
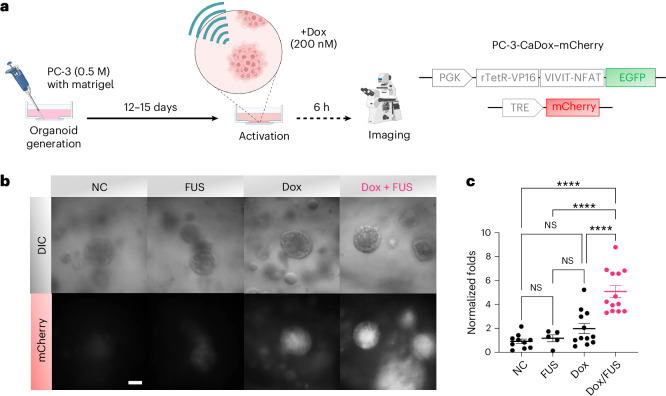
FUS-based mechanogenetic activation in a 3D organoid model. **a**, Schematic illustration of the experimental workflow showing PC-3-CaDox–mCherry cell-derived organoid formation, FUS stimulation (2 MHz, 1 min on/4 min off, six cycles), doxycycline (200 nM) treatment and mCherry imaging 6 h post-activation. **b**, Representative differential interference contrast and mCherry fluorescence images of organoids under four conditions: no treatment (NC), FUS only, Dox only and combined Dox + FUS. Scale bar, 50 µm. Images are representative of ≥5 independent experiments. **c**, Quantification of mCherry fluorescence intensity in individual organoids from the four treatment groups: NC (*n* = 10), FUS only (*n* = 5), Dox only (*n* = 12) and Dox + FUS (*n* = 13). Each dot represents one organoid (biological replicate). Data are presented as mean ± s.e.m. Statistical significance was determined using ordinary one-way ANOVA with Tukey’s multiple comparisons test (two-sided). Adjusted *P* values are as follows: NC versus Dox + FUS, *P* < 0.0001; FUS versus Dox + FUS, *P* < 0.0001; Dox versus Dox + FUS, *P* < 0.0001; all other comparisons were not statistically significant (*P* > 0.28). **** denotes adjusted *P* < 0.0001. Panel **a** created with BioRender.com. Source data

Next we evaluated the functionality of the CaDox system in controlling genetics upon FUS stimulation. We engineered PC-3-CaDox cell lines with mCherry as a reporter gene to generate 3D organoids^23,30^, which were subjected to treatment with both doxycycline and FUS (Fig. 3a). Specifically, organoids were pre-incubated with 200 nM doxycycline, followed by repetitive FUS stimulations for 30 min (1 min on and 4 min off, six times) using a 2 MHz transducer (peak negative pressure, 0.63 MPa) with pulsed waves (duty cycle, 10%) to activate the CaDox system (Fig. 3a). The imaging results revealed that neither FUS nor doxycycline alone resulted in a significant gene induction (Fig. 3b,c). However, when both stimuli were applied, the CaDox system led to a clear and significant gene induction compared with all other groups (*P* < 0.0001). These results showed that the mechanical stimulation by FUS can be transduced by the CaDox system to control designed gene expressions in cancer cells.

## FUS activates CaDox for spatial gene control in vivo

**Fig. 4:**
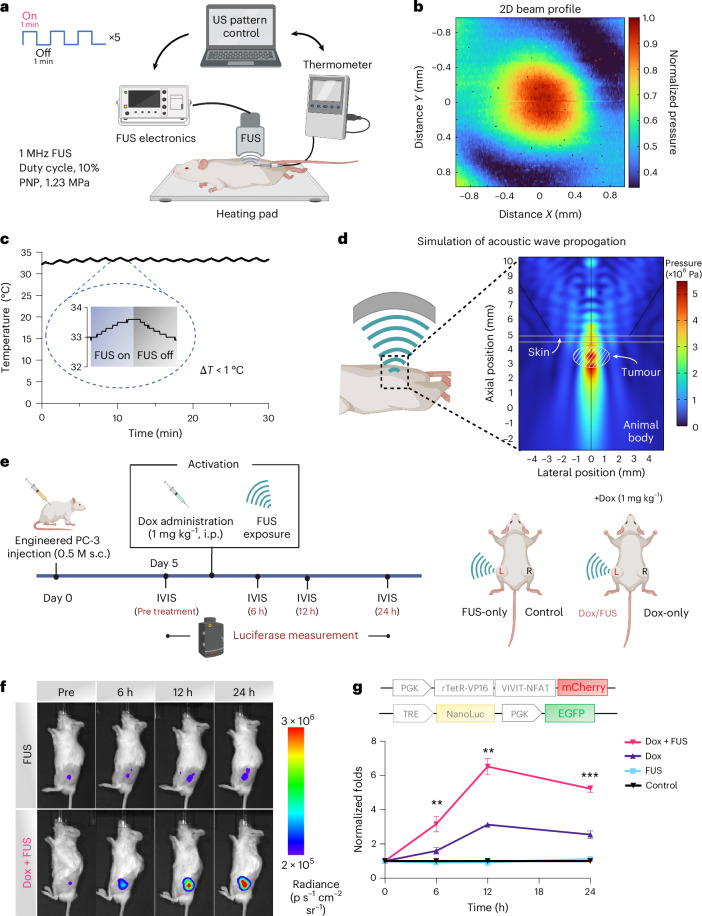
In vivo characterization of the FUS-based mechanogenetic system. **a**, Schematic of the custom-built FUS stimulation set-up for small animals. A Python-controlled user interface was used to define stimulation patterns and parameters, which directed a function generator and power amplifier to drive the FUS transducer. Temperature changes in the tumour region were monitored by a needle-type thermometer during the 30 min stimulation window. PNP, peak negative pressure. **b**, Two-dimensional beam profile of the 1 MHz transducer at the focus, measured by hydrophone. **c**, Temperature recordings at the tumour site during FUS stimulation, showing fluctuations of less than 1 °C. **d**, Simulated acoustic wave propagation and spatial distribution of mechanical stress in a layered mouse tissue model, including skin (0.5 mm thick), muscle and a 3 × 3 × 1.8 mm^3^ embedded tumour. **e**, Experimental design of in vivo activation of the CaDox system by FUS. PC-3-CaDox–NLuc cells were injected into NSG mice to establish a bilateral tumour model. On day 5, tumours were treated with either doxycycline, FUS or both. Bioluminescence imaging (BLI) was used to monitor luciferase intensities over a 24 h period. L, left; R, right; s.c., subcutaneous; IVIS, in vivo imaging system. **f**, Representative BLI images of gene expression in subcutaneous tumours in response to the treatment with FUS alone or combined doxycycline and FUS. Images are representative of four mice per group. p, photons. **g**, Quantification of normalized gene expression fold change over time (*n* = 4 mice per group). Signal intensities were normalized to the untreated control. Error bars indicate s.e.m. Statistical significance was determined using a two-sided paired *t*-test. Adjusted *P* values comparing Dox only versus Dox + FUS conditions were as follows: 6 h, *P* = 0.0098; 12 h, *P* = 0.0033; and 24 h, *P* = 0.0003. ** and *** denote adjusted *P* < 0.01 and *P* < 0.001, respectively. Panels **a**, **d** and **e** created with BioRender.com. Source data

To examine the FUS-CaDox system and mechanogenetics in vivo, we performed FUS stimulation experiments using engineered PC-3-CaDox cells in a xenograft mouse model. We designed a custom FUS stimulation apparatus in-house (Fig. 4a and Supplementary Fig. 8) featuring a 1 MHz focused transducer (diameter, 70 mm; radius of curvature, 65 mm) to deliver targeted mechanical stimulation to a localized volume at the tumour site (Fig. 4b and Supplementary Fig. 9). FUS parameters are chosen to generate robust mechanical perturbations while maintaining the acoustic power exerted on the targets as in 2D and organoid studies, as well as maintaining safety thresholds and avoiding non-specific tissue disruptions. As such, the FUS power (peak negative pressure, 1.23 MPa) and repetition frequency (1 min intervals every 2 min over a 30 min period; Fig. 4a) were employed to maintain the mechanical index below the US Food and Drug Administration (FDA) safety guideline of 1.9 and avoid the risk of cavitation effects^31^. This setting also allows us to control the thermal output of the system so that the temperature variation did not exceed 1 °C (Fig. 4c), circumventing potential non-selective thermal effects that could confound our results. Overall, this approach allowed us to precisely explore the genetic control of mechanical stimulation using FUS in an in vivo setting while adhering to safety guidelines.

To gain a more comprehensive understanding of acoustic wave propagation within the animal body and to estimate the amount of energy deposited at the focus of the ultrasound, we conducted simulations using the COMSOL Multiphysics software package to assess the spatial distribution of acoustic wave pressure^32,33^. Our findings revealed a minimal difference in the pressure peak and distribution of the FUS, when accounting for the attenuation due to the animal’s skin and muscle^32,33^ (Supplementary Fig. 10). Our results also demonstrated that FUS can generate a focal pressure distribution covering an area of approximately 1.5 mm in the *x* or *y* direction and 2 mm in the *z* direction (Fig. 4d).

We next engineered PC-3-CaDox cell lines with an inducible NanoLuc luciferase (NLuc) reporter and implanted them into NOD scid gamma (NSG) mice to establish a bilateral tumour model (Fig. 4e). Five days post-implantation, we divided the mice into two groups. One group received doxycycline (1 mg kg^−1^) while the other group was left untreated. FUS stimulation was then applied to the left tumours of mice in both groups, leaving the right tumours as unstimulated controls for reference. We monitored the NLuc expression levels before treatment, as well as at 6, 12 and 24 h post-doxycycline administration, to track the temporal gene induction dynamics of the CaDox system activation.

Significant gene expression was observed only in the group receiving the combination of doxycycline and FUS treatment (left tumour; Fig. 4f,g and Supplementary Fig. 11). No induction of gene expression was observed in the non-treated control group nor in the group with FUS stimulation alone, in the absence of doxycycline. A minor and statistically insignificant induction of gene expression can be observed in the control group with doxycycline-only treatment. No signs of tissue damage or alteration in tumour growth were observed at the FUS-treated sites. These results underscore the safety and applicability of the FUS-CaDox system for in vivo control of designed gene expressions.

## Mechanogenetic control of combinatorial immunotherapy

**Fig. 5:**
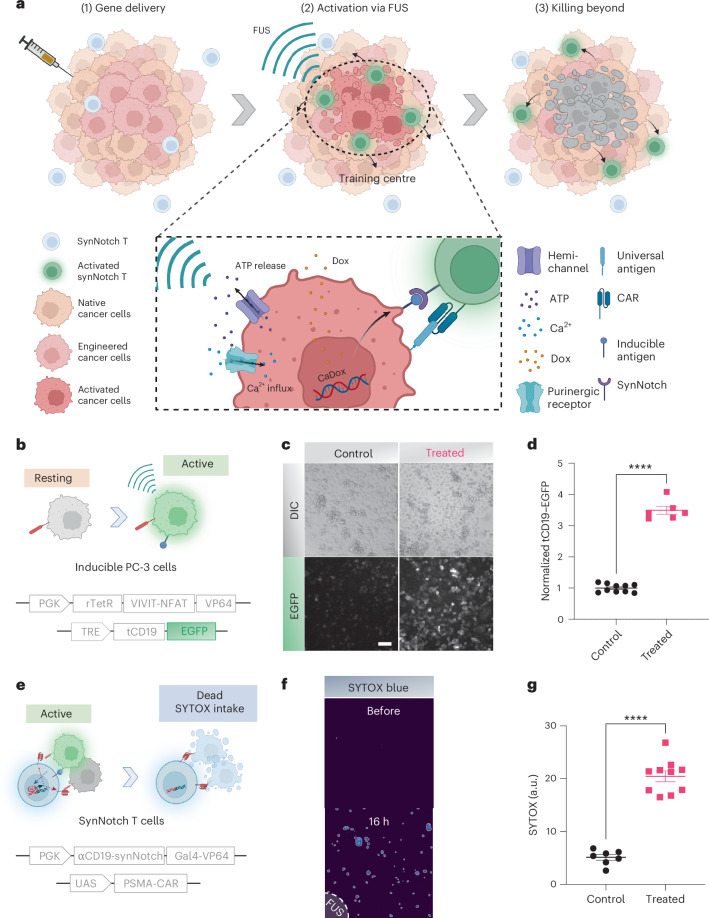
Combinatorial immunotherapy in vitro*.* **a**, Schematic diagram of the combinatorial immunotherapy strategy. First, tumours are transduced with the CaDox genetic system. Upon localized FUS stimulation, the CaDox system is activated in a subset of tumour cells, leading to ATP release via PANX1 hemichannels and calcium influx through purinergic receptors. This results in the expression of clinically validated antigens, such as tCD19, which prime synNotch CAR T cells (synNotch T). Activated synNotch CAR T cells then express anti-PSMA CAR to target and eliminate surrounding tumour cells. **b**, PC-3 cells were equipped with the CaDox–tCD19–EGFP system to express tCD19 and EGFP upon FUS activation. **c**,**d**, Representative images of induced gene expression (**c**) and quantified EGFP expression (**d**) levels measured in no treatment (*n* = 10) or combined treatment (*n* = 6) groups. Data represent mean ± s.e.m. Statistical significance was determined by unpaired two-tailed Welch’s *t*-test; adjusted *P* < 0.0001. **e**, Primary T cells were engineered with anti-CD19 (αCD19) synNotch and inducible anti-PSMA CAR to be activated by the induced tCD19^+^ cells to attack and clear PSMA^+^ cancer cells. UAS, upstream activating sequence (Gal4-binding promoter); SynNotch T, synthetic Notch T cells. **f**, Representative images of SYTOX staining to assess the cytotoxicity of PC-3 cells. Dashed line indicates the FUS-targeted region. **g**, Quantification of cytotoxicity of PC-3 cells in no treatment (*n* = 7) and treatment (*n* = 10) groups. Data represent mean ± s.e.m. Statistical significance was determined by unpaired two-tailed Welch’s *t*-test; adjusted *P* < 0.0001. Images in **c** and **f** are representative of ≥3 independent experiments. **** denotes adjusted *P* < 0.0001. Panels **a**, **b** and **e** created with BioRender.com. Source data

We then applied our FUS-CaDox mechanogenetic system for tumour priming in immunotherapy, integrating with synNotch and CAR T cells^34^. The FUS-CaDox is rewired to produce a clinically validated and specific antigen CD19 upon the doxycycline-gated FUS stimulation. This induced subpopulation of tumour cells expressing CD19 can then serve as priming cells and local ‘training centres’ to stimulate the subsequently infused CD19–synNotch CAR T cells, which can be activated through a synNotch receptor recognizing CD19 to produce a CAR against a less specific but widespread tumour antigen expressed on tumour cells to eradicate the whole tumour population at the tumour site and its neighboring regions (Fig. 5a). Therefore, the FUS-CaDox priming of tumour cells integrated with the killing by synNotch CAR T cells can overcome these problems: (1) the lack of clinically validated antigens for solid tumours; (2) the low and varying gene delivery efficiency in vivo; and (3) the limitations in spatial coverage of FUS.

To validate the concept of the combinatorial immunotherapy, we first generated PC-3-CaDox cell lines with the CaDox reporter encoding an inducible truncated CD19 (tCD19), which has been clinically validated to serve as a specific and efficient target for CAR T therapy^35^ (Fig. 5b). We fused EGFP to the cytoplasmic tail of tCD19 and verified that tCD19 retains plasma membrane localization by imaging, and confirmed that the extracellular epitope of tCD19 can be recognized by anti-CD19 antibody (Supplementary Fig. 12). A combined treatment of doxycycline and FUS triggered significant tCD19 expression on the cell membrane (Fig. 5c,d and Supplementary Fig. 13).

After confirming the tCD19 inducibility of the cancer cells, we genetically engineered Jurkat T cells with an anti-CD19 synNotch receptor to drive TagBFP (BFP, blue fluorescent protein) as an activation reporter for synNotch CAR T (Supplementary Fig. 14a). The activation of Jurkat T cells, as indicated by TagBFP, was significantly higher when they were co-cultured for 24 h with PC-3 cells pre-activated by the doxycycline-gated FUS to express tCD19 (Supplementary Fig. 14b). This result indicates that tCD19 induced by FUS-CaDox can train and lead to synNotch activation and reporter production in T cells.

We then examined the synNotch-mediated killing using primary human T cells. For the homologous antigen broadly expressed in prostate cancer cells, we chose prostate-specific membrane antigen (PSMA) and introduced an inducible anti-PSMA CAR in the synNotch CAR T cells (Fig. 5e). Flow cytometry analysis showed robust activation of these CD19/synNotch-PSMA-CAR (synNotch-CAR) T cells when co-cultured with PC-3 cells under active conditions compared with resting conditions (Supplementary Fig. 14c,d), confirming that synNotch-PSMA-CAR expression can be effectively triggered by interaction with inducible tCD19. To characterize the cytotoxicity and specificity of the synNotch-CAR T cells, we first generated three different PC-3 cell lines, each constitutively expressing either tCD19, PSMA or both tCD19 and PSMA. Each of these PC-3 cell lines was co-cultured with the synNotch-CAR T cells (effector-to-target ratio, E/T = 1:1) for 24 h before assessing the killing efficiency. Without the PSMA antigen, no significant cytotoxicity was observed (Supplementary Fig. 15a). Minor cytotoxicity was observed in the CD19^−^/PSMA^+^ group (*P* = 0.0419), but significant killing was found only in the CD19^+^/PSMA^+^ group (*P* < 0.0001; Supplementary Fig. 15a), demonstrating that our synNotch-CAR T cells can specifically recognize and kill cells expressing both CD19 and PSMA. Next we examined whether the expression of tCD19 on a subpopulation of cancer cells could lead to the killing of the whole tumour population with a homologous PSMA antigen. We activated the CaDox-inducible tCD19 in PSMA-expressing PC-3 cells to achieve an approximate 15% tCD19^+^ subpopulation (Supplementary Fig. 15b). When we co-cultured these mixed tCD19^+^^/^^−^ cells with synNotch-CAR T cells, the fraction of viable cells dropped to 0.56 within 24 h and 0.24 within 48 h at an E/T ratio of 1:1, in contrast with the non-affected vial fraction of pure CD19^−^/PSMA^+^ PC-3 cells (Supplementary Fig. 15b). This result suggests that once trained and activated by the inducible tCD19, synNotch-CAR T cells can kill adjacent tumour cells expressing PSMA, no longer dependent on tCD19 (refs. ^34,36^). SYTOX blue staining consistently showed a significant killing of the cancer cells in the neighbouring regions in and outside of the area exposed to doxycycline-gated FUS stimulation (Fig. 5f,g).

## In vivo examination of spatially guided CAR T therapy

Next we examined the tumour priming effect of the FUS-CaDox mechanogenetic system integrated with synNotch-CAR T cells for cancer immunotherapy in an in vivo model. We followed the same experimental design and FUS parameters that we used for the in vivo FUS-CaDox gene inducibility testing (Fig. 4). Specifically, we first injected NSG mice on each side of each mouse with 0.5 million PSMA^+^/FLuc^+^ PC-3-CaDox cells expressing inducible tCD19 to establish bilateral tumour models. We continuously monitored tumour growth using bioluminescence measurements starting from day 5. On day 10, we separated the mice into two groups, with or without the intraperitoneal (i.p.) doxycycline administration (1 mg kg^−1^), and stimulated all mice on the left-side tumours with only FUS (Supplementary Fig. 16a). No statistical difference in tumour growth was observed between the FUS-CaDox-primed and control groups without the introduction of synNotch-CAR T cells (Supplementary Fig. 16b,c). When synNotch CAR T cells (0.5 million per tumour site) were administered subcutaneously right after FUS treatment on day 10 (Supplementary Fig. 17a), tumour growth in the doxycycline-only or FUS-only group was not statistically different from that of the negative control group without any treatment (Supplementary Fig. 17b,c). By contrast, synNotch-CAR T cells can significantly suppress the FUS-CaDox-primed tumour growth (Supplementary Fig. 17b,c).

We then investigated the in vivo gene delivery of our CaDox circuit into pre-existing tumours via viral vectors to translate our findings into a more clinically relevant model. We first evaluated the kinetics and biodistribution of viral-mediated gene expression following focal administration of the AAV2 vector expressing FLuc, peritumourally into NSG mice with pre-existing PC-3 tumours. Although the initial FLuc expression was localized to the tumour site, clear spread to adjacent tissues, including the underlying muscle, was observed by day 30 (Supplementary Fig. 18a). This result demonstrates that even locally injected viral vectors can leak into adjacent regions, resulting in prolonged and uncontrolled gene expression after injection. This highlights the potential benefit of using FUS to provide precise spatial and temporal control over gene expression, thereby improving safety and therapeutic outcomes.

**Fig. 6:**
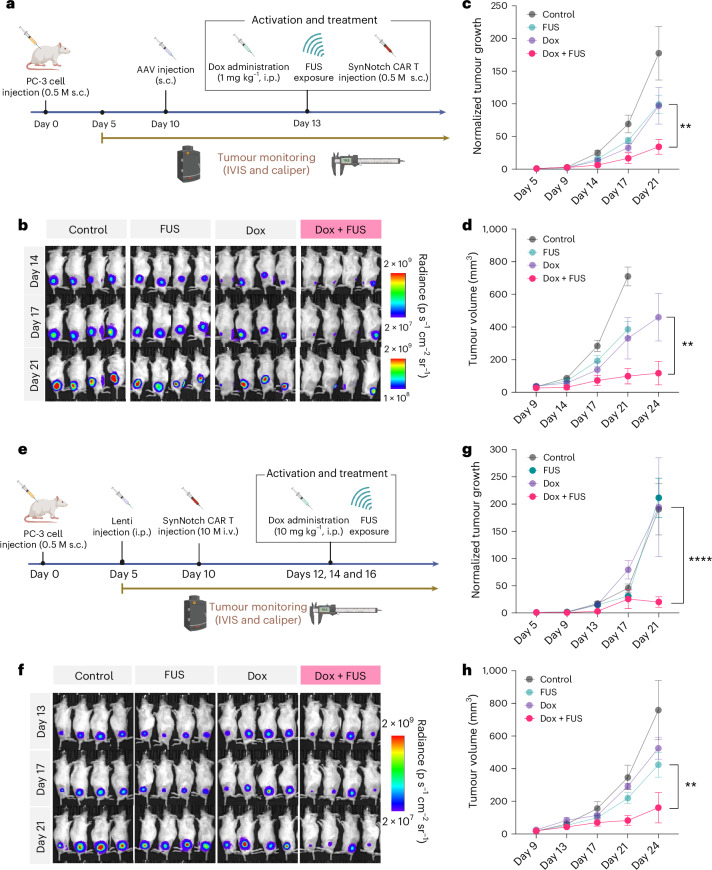
Combinatorial immunotherapy in vivo using viral-vector-mediated CaDox delivery. **a**,**e**, Schematic representation of experimental timelines for two different gene and T cell delivery approaches used to evaluate the therapeutic efficacy of the FUS controllable cell immunotherapy. In **a**, NSG mice with bilateral PC-3 tumours received peritumoural AAV-mediated CaDox delivery, followed by local FUS activation and peritumoural T cell administration. In **e**, an alternative strategy employed systemic lentiviral delivery of the CaDox circuit, followed by local FUS activation and systemic T cell administration. i.v., intravenous. **b**,**f**, IVIS imaging of tumour bioluminescence at multiple time points to compare tumour burden across treatment groups. In **b**, mice received local AAV-CaDox injection, whereas in **f**, mice received systemic injections of lentiviral CaDox constructs and CAR T cells. **c**,**g**, Quantification of luciferase signal intensity normalized to pretreatment levels over time. Adjusted *P* values at the end-point are as follows: in **c**, *P* = 0.0042; in **g**, *P* < 0.0001. **d**,**h**, Tumour volume measurements over time using caliper-based assessments. Adjusted *P* values at end-point are as follows: in **d**, *P* = 0.0076; in **h**, *P* = 0.0037. Each line represents the group mean ± s.e.m. (*n* = 4 mice per group). Statistical significance was determined using two-way ANOVA with Tukey’s multiple comparisons test (two-sided). ** and **** denote adjusted *P* < 0.01 and *P* < 0.0001, respectively. Panels **a** and **e** created with BioRender.com. Source data

We next explored the therapeutic efficacy of our approach via viral delivery of the CaDox system peritumourally into NSG mice with pre-existing PC-3 tumours, followed by doxycycline administration and FUS exposure to activate the CaDox circuit before the local introduction of synNotch CAR T cells (Fig. 6a). Tumour progression data demonstrated that the combined doxycycline and FUS treatment effectively primed the tumour cells, resulting in significant suppression of tumour growth compared with the control groups (Fig. 6b–d). These results suggest that our approach is effective with the local viral gene delivery into pre-existing tumours in vivo.

To determine whether our therapeutic approach could be applied in scenarios where focal injection is not feasible, we evaluated the potential of systemic delivery of the CaDox circuits. Using the AAV2–CMV–FLuc virus, we found that i.p. administration resulted in the delivery of the viral vector to the tumour site, along with widespread distribution across multiple organs, including the liver, spleen, bladder and testis (Supplementary Fig. 18b,c). NSG mice with pre-existing PC-3 tumours were then administered with viral vectors containing the CaDox circuit systemically (i.p.) on day 5, followed by tumour priming by doxycycline-gated FUS and subsequently synNotch CAR T administration on day 12 (Supplementary Fig. 19a). The doxycycline-gated FUS treatment resulted in a marked reduction in tumour growth compared with the untreated group (Supplementary Fig. 19b–d). Histopathological analysis was further conducted on major organs, including the brain, heart, kidney, liver and lung (Supplementary Fig. 20). No clear off-tumour toxicity or tissue damage was observed in the treated group, indicating that the systemic administration of the CaDox circuit, combined with FUS-mediated activation, can be a safe strategy.

We further examined the systemic administration of synNotch CAR T cells. NSG mice with bilateral PSMA^+^/FLuc^+^ PC-3-CaDox tumours were administrated with synNotch CAR T cells (10 million per mouse) intravenously on day 8 (Supplementary Fig. 21a). To allow time for the systemically introduced synNotch CAR T cells to reach the tumour sites and maximize the tumour priming effect, the doxycycline-gated FUS tumour priming was applied multiple times (days 10, 12 and 14) after synNotch CAR T administration. This resulted in notable tumour suppression in the doxycycline-gated FUS priming group, compared with the control (doxycycline only; *P* = 0.0004; Supplementary Fig. 21b–d).

Lastly we conducted a complete treatment that integrates systemic delivery of both the CaDox gene circuit and synNotch CAR T cells, while applying FUS targeted to the local tumour site, to explore the feasibility of our combinatorial therapy in a more clinically relevant setting. NSG mice with pre-established bilateral PSMA^+^/FLuc^+^ PC-3 tumours were administered lentiviral vectors containing the CaDox circuit via systemic injection (i.p.), followed by FUS-mediated activation and systemic administration of synNotch CAR T cells (Fig. 6e). This approach resulted in effective tumour priming, leading to significant tumour suppression upon treatment (Fig. 6f–h). These findings demonstrate the flexibility of the FUS-CaDox system in controlling gene expression and achieving tumour suppression through systemic administration of both the gene circuit and synNotch CAR T cells, further expanding the potential applicability for tumour-targeted immunotherapy.

## Outlook

In this study we developed a FUS-based mechanogenetics approach without the need of co-factors such as microbubbles, aiming to reveal the translational potential of direct, remote and non-invasive control of cellular mechanics and designed genetics using ultrasound. To achieve this, we exploited the mechanosensitivity of cancer cells and rewired the FUS-mediated calcium responses to user-defined transcriptional activity using the calcium-dependent and doxycycline-dependent AND-logic-gated circuit, referred to as the CaDox system. This approach allowed us to apply FUS for remote and non-invasive genetic priming of solid tumours for synNotch-CAR T treatment. The resulting subpopulation of tumour cells expressing a clinically validated antigen upon doxycycline-gated FUS stimulation can serve as priming cells and local ‘training centres’ for the activation of synNotch CAR T cells, which will then, at the local neighbourhood of the tumour site, attack the whole population of cancer cells expressing a less specific but widespread tumour antigen, and eradicate the tumour.

For potential clinical translation, we envision gene delivery via systemic administration of viral vectors (for example, intravenously), followed by FUS stimulation targeted to tumour regions using MRI^37^ or ultrasound^38^ guidance, depending on the anatomical location and available clinical infrastructure (more information is in Supplementary Note). Similarly, synNotch CAR T cells could be administered systemically, with tumour-localized FUS activation serving as the spatial cue to restrict immune activation. While this study focused on subcutaneous tumour models, future optimization efforts will be required to adapt this approach to internal tumours, including in the brain, where FUS technologies are being actively developed but still face practical constraints such as imaging access and regulatory approval for combination therapies. Finally, although our combinatorial immunotherapy relies on FUS to effectively target deep tumours, clinical success also depends on efficient gene delivery. Advances in viral vector engineering^39^, nanoparticle-based carriers^40^ and FUS-mediated blood–brain barrier opening^41^ are actively improving gene therapy accessibility, broadening the applicability of mechanogenetic approaches. As these technologies evolve, our approach could be integrated synergistically with existing treatments, expanding therapeutic options for resistant tumours and advancing more precise, adaptable cancer therapies.

## Methods

### Study design and statistical analysis

This study was designed to validate mechanogenetic control strategies using FUS in both in vitro and in vivo models. For non-animal experiments, each condition was tested using at least three independent biological replicates. For in vivo experiments, a minimum of four mice per group was used to ensure reproducibility while minimizing animal use. Sample sizes were not predetermined by statistical methods but were based on prior literature^5^ and standard practice in the field. Mice were randomly assigned to treatment groups. No formal blinding was performed; data collection and analysis were not blinded to group allocation. Data distribution was assumed to be normal, but this was not formally tested. No animals or data points were excluded from analysis. Statistical comparisons were performed using unpaired two-tailed Welch’s *t*-test or one-way ANOVA with Tukey’s multiple comparisons test, as indicated in the figure captions. Two-way ANOVA was used for time-course data. The detailed statistical test methods are indicated in the corresponding figure captions. Flow cytometry data were analysed using FlowJo v.10.8.2, and all other statistical analyses and visualizations were performed using GraphPad Prism v.10.0.0. Selected illustrative schematics were created using BioRender under an academic license.

### Animals

All animal procedures were conducted in accordance with relevant ethical regulations and were approved by the Institutional Animal Care and Use Committee (IACUC) at both the University of California, San Diego (UCSD, protocol S15285) and the University of Southern California (protocol 21479). All researchers involved in animal work adhered to institutional guidelines throughout this study. For all animal experiments, male NOD.Cg-Prkdcscid Il2rgtm1Wjl/SzJ (NSG) mice (6–8 weeks old) were used. Mice were obtained from the UCSD Animal Care Program or the Jackson laboratory (005557). The animals were housed under a 12 h light/12 h dark cycle, with an ambient temperature of 20–24 °C and relative humidity maintained between 40–60%. The maximum allowable tumour size under both institutional protocols was 1.5 cm in diameter or approximately 10% of body weight. Tumour growth was closely monitored, and no animals exceeded these limits throughout the study.

### Cloning

Plasmids were generated using either the Gibson Assembly (New England Biolabs, E2611L) method or the T4 DNA ligation (New England Biolabs, M0202L) method, depending on the specific requirements of each construct. Throughout the study, the appropriate cloning strategy was employed for each construct, and the resulting plasmids were used for subsequent experiments as described in the relevant sections of Methods. All plasmids used in this study are detailed in Supplementary Table 1.

### Site-directed mutagenesis of NFAT for variant calcineurin binding sequences

To elucidate the kinetic differences associated with varying affinities to calcineurin, we engineered several NFAT variants, each harbouring distinct calcineurin binding sequences. These modifications were introduced using the Q5 Site-Directed Mutagenesis Kit (New England Biolabs, E0554S), following the manufacturer’s instructions. The primers for the mutagenesis processes were designed via the NEBaseChanger website (https://nebasechanger.neb.com), ensuring both specificity and efficiency. Following mutagenesis, the derived NFAT variants underwent Sanger sequencing for verification, confirming the intended modifications in the calcineurin binding sequences.

### Cell culture and reagents

The cell lines used in this study, including HEK293T (CRL-3216), PC-3 (CRL-1435), Jurkat T (TIB-152), U-87MG (HTB-14) and MDA-MB-231 (CRM-HTB-26), were obtained from the American Type Culture Collection (ATCC). All cell lines were authenticated by ATCC using short tandem repeat profiling and were used within early passages upon receipt. The Lenti-X 293T cell line (Clontech, 632180) was purchased from Takara Bio, which provides authenticated and validated cells. HEK293T, PC-3, U-87MG and MDA-MB-231 cells were cultured in Dulbecco’s Modified Eagle Medium (DMEM; Gibco, 10569069) supplemented with 10% heat-inactivated foetal bovine serum (FBS; Gibco, 16140071) and 1% penicillin–streptomycin (Gibco, 15140122). Jurkat T cells were maintained in Roswell Park Memorial Institute Medium (RPMI-1640, Gibco, 22400105) supplemented with 10% heat-inactivated FBS and 1% penicillin–streptomycin. Primary T cells were cultured in X-Vivo 15 medium (Lonza, BE02-060Q) supplemented with 5% heat-inactivated FBS, 50 μM 2-mercaptoethanol (Gibco, 31350010) and 1% recombinant human interleukin-2 (Peprotech, 200-02). All cells were maintained in a humidified incubator at 37 °C with 5% CO_2_ and passaged as per standard protocols to ensure optimal growth and experimental conditions. All cell lines were routinely tested and confirmed negative for mycoplasma contamination.

### Exogenous gene delivery and establishment of stable cell lines

To investigate the kinetics of NFAT variants in HEK293T cells, we used a Lipofectamine 3000 kit (Thermo Fisher, L3000015) to transiently deliver exogenous genes into cells, following the manufacturer’s guidelines. Briefly, we mixed the Lipofectamine reagent with DNA plasmids encoding the exogenous genes of interest and incubated the mixture at room temperature for 15 min to allow complex formation. We then added the complex to the cells and incubated them for 24–48 h before performing time-lapse imaging.

To engineer cell lines for stable expression of exogenous genes, such as CaDox components, we used viral transduction based on lentiviral methods. Briefly, we produced lentiviral particles by co-transfecting Lenti-X packaging cells with the transfer vector encoding the gene of interest, along with packaging plasmids (pVSV-G and pΔR) using the calcium phosphate method (Promega, E1200). After 48 h, the supernatant containing the lentiviral particles was collected, filtered through a 0.45 µm filter and concentrated using Lenti-X Concentrator (Takara, 631232). The concentrated viral pellet was resuspended in 100 µl of phosphate-buffered saline (PBS) and stored at −80 °C until further use. For transduction, target cells were seeded in six-well plates at a density of 0.1 million cells per well. The following day, the cells were transduced with the concentrated lentiviral particles. Transduced cells were subsequently selected for stable expression using fluorescence-activated cell sorting (Sony, SH800) to establish a cell population stably expressing the gene of interest.

### Primary T cell isolation and transduction

Peripheral blood mononuclear cells were obtained from buffy coats provided by the San Diego Blood Bank and isolated using a lymphocyte separation medium (Corning, 25-072-CV), following the manufacturer’s guidelines. Primary human T cells were then extracted from the peripheral blood mononuclear cells using the Pan T Cell Isolation Kit (Miltenyi, 130-096-535) and activated with Dynabeads Human T-Expander CD3/CD28 (Gibco, 11141D) on the same day. For transduction, concentrated lentivirus was added to the T cells on day 3 at a multiplicity of infection of 10 and subjected to spinoculation in a 24-well plate coated with 30 µg ml^−1^ RetroNectin (Takara, T100B). T cells continued to expand, and Dynabeads were removed on day 6. When required, transduced T cells were sorted using a fluorescence-activated cell sorting machine (Sony, SH800) to acquire a pure population.

### Fabrication and characterization of ultrasonic transducers

For the in vitro study, we fabricated two ultrasound transducers in-house using standard methods described previously^42^. The first was a lithium niobate (LiNbO_3_) pressed-focused 35 MHz transducer (f-number = 1, focal length = 6 mm). Higher frequency transducers offer advantages for mechanistic studies. A higher frequency results in a narrower focused area, which allows precisely localized stimulation. Moreover, as the mechanical response of cells to ultrasound is often more pronounced at higher frequencies, less power is required to induce cellular responses. This is related to the frequency-dependent nature of ultrasound interaction with biological tissues, where higher frequencies exhibit greater acoustic attenuation.

We also designed and fabricated a highly focused single-element 2 MHz ultrasound transducer (f-number = 0.67, focal length = 6 mm). This transducer was used to examine cellular responses at a frequency that is clinically relevant, providing important insights for potential therapeutic applications. Modified hard lead zirconate titanate (PZT; Del Piezo, DL-47) was used for this transducer, which is known for its high-power handling capability.

For the animal study, we constructed a focused 1 MHz single-element transducer in-house using a prefocused modified PZT (diameter, 70 mm; radius of curvature, 65 mm; DL-47, Del Piezo) with a 20 mm hole in the centre based on the design from a previous report^43^.

Transducer characterization was conducted by measuring the axial and lateral resolution, as well as intensities per applied voltage, using a needle hydrophone (Onda, HGL-0085; Fig. 4 and Supplementary Figs. 1 and 9). The peak negative pressures and spatial-peak, temporal-average intensity (*I*_SPTA_) served as indicators of the strength of the FUS used in each experiment.

### In vitro ultrasound stimulation

For in vitro studies, we used a FUS stimulation set-up integrated with an inverted epifluorescence microscope. We used press-focused 35 MHz and 2 MHz FUS single-element transducers, which were manipulated by a 3D linear stage controller (Sigma Koki, SHOT-304GS) for precise position control. User-defined input signals were generated using a function generator (Stanford Research Systems, SG380) and were amplified by a 50 dB power amplifier (E&I, 325LA) before being fed into the ultrasonic transducers. To position the cells at the transducer’s axial focus, we employed a pulse-echo method using a pulser receiver (Olympus, 5072PR) and oscilloscope (Teledyne Lecroy, 610Zi) to determine the distance between the transducer and culture dish that yielded the maximum echo signal. To align the transducer’s lateral focus with the centre of the CCD (charge-coupled device) camera of the microscope, we used an acoustic trapping method described previously^44^.

Other detailed in vitro experimental procedures, including protocols for CRISPR-mediated gene knock-out, calcium imaging, temperature sensing, luciferase and fluorescence-based assays, protein detection, 3D cell culture and viral vector biodistribution studies, are provided in Supplementary Methods.

### In vivo ultrasound apparatus and stimulation

We developed an in-house FUS stimulation system for animal studies using a 1 MHz transducer to induce localized mechanical perturbation. A coupling cone (length, 65 mm) with a 4 mm diameter opening at the tip was designed and 3D printed to hold degassed water along the acoustic path to the animals (Supplementary Fig. 8). The opening at the end was sealed with an acoustically transparent Mylar film (thickness, 2.5 µm; Chemplex, 100) to prevent water leakage. Custom Python code was written to control a function generator (Stanford Research System, SG386) by sending user-defined input parameters and repetition patterns to the generator. The signal was then amplified by a 50 dB power amplifier (E&I, 325LA) before being fed to the transducer. A set of manual translational stages were employed to control the transducer’s focus. Additionally, a feedback-controlled temperature pad (Auber Instruments, WSD-30B) was used to warm the animal bed to maintain each animal’s body temperature under anaesthesia. An acoustic absorber (Precision Acoustics, Aptflex F28) was placed under the animal to reduce any reflections.

For the stimulation procedure, doxycycline (1 mg kg^−1^) was first injected into mice 5 to 7 min before anaesthesia to allow sufficient circulation in the body. The anaesthetized mouse was then placed on the animal bed with its left side on top, and acoustic gel (Aquasonic, 26354) was applied to the tumour area as a coupling media that fills potential air gaps to maximize the transmission of FUS. If necessary, the region was shaved using a handheld trimmer before gel application. The 1 MHz transducer was positioned over the marked tumour area with the tip of the coupling cone placed within 0.5 mm of the skin.

For real-time thermometry, a needle-type thermocouple (29 gauge; time constant, 0.25 s; MT-29/2HT, Physitemp Instruments) with a thermometer (HH806AU, Omega) was used. During the procedure, the tip of the needle-type thermocouple was inserted subcutaneously from the side into the mouse near the target region, while the focused transducer was placed on top of the tumour to stimulate the area.

### Acoustic wave simulation

The simulations were performed using COMSOL Multiphysics v.6.0 software and the finite element method, a numerical technique for solving partial differential equations by discretizing the domain into smaller elements. In our study, the frequency-domain pressure acoustic module within COMSOL was employed to simulate the ultrasound field, as this module is specifically designed to analyse pressure wave propagation and interactions in various media. To ensure the accuracy and reliability of the simulations, material parameters related to ultrasound, such as density, acoustic impedance and attenuation, as well as animal body properties like skin thickness, normal tissue characteristics and tumour tissue properties, were sourced from relevant literature^45–49^. This approach allowed us to create a more realistic representation of the materials and conditions in our simulations, ultimately leading to a better understanding of the acoustic wave propagation within the animal body and the amount of energy deposited at the focus of the ultrasound.

### Cell viability assay

To access cell viability in spheroids following FUS treatment, a LIVE/DEAD Cell Imaging Kit (Invitrogen, R37601) was used, following the manufacturer’s instructions. U87 spheroids were first prepared and exposed to 2 MHz FUS under the same conditions used for CaDox activation (Supplementary Fig. 1b). The apoptotic reagent paclitaxel (200 nM) was used as a positive control for cell death. Fluorescence images were acquired before stimulation (0 h) and after 72 h, and spheroid viability was quantified based on the dead cell signal.

To visualize and assess synNotch T cell-mediated killing of antigen-inducible cancer cells in vitro, SYTOX blue dead cell stain (Thermo Fisher, S34857) was used. SYTOX blue dead cell stain is a cell-impermeant nucleic acid dye that selectively binds to the DNA of dead cells with compromised cell membranes, enabling the visualization and quantification of cell death in real-time imaging experiments. Engineered PC-3-CaDox–tCD19–PSMA^+^ cell lines were seeded onto 35 mm culture dishes 24 h before stimulation. On the following day, the seeded cells were treated with or without doxycycline and then subjected to FUS stimulation at designated locations on the dishes. After removing the drug with Dulbecco’s PBS, synNotch T cells were added to the dishes in media containing 25 nM SYTOX. Time-lapse images were captured every 5 min for a duration of 24 h to monitor the viability of the PC-3 cells.

### In vivo bioluminescence imaging

To validate the inducibility of the CaDox system against FUS stimulation as well as to monitor the tumour growth in animals, in vivo BLI was conducted using an IVIS Lumina LT Series III (PerkinElmer). For NLuc imaging, Nano-Glo In Vivo Substrate (Promega, FFz, CS320501) was i.p. injected at a volume of 100 µl of reconstituted solution (0.44 micromoles), following the manufacturer’s guidelines. Imaging commenced 5 min after injection and continued until the peak signal was detected. For FLuc imaging, d-luciferin (GoldBio, LUCK) was i.p. administered at a dose of 150 mg kg^−1^. BLI began 10 min post substrate injection and continued until the maximum signal was reached. Living Image software (PerkinElmer) was employed for image analysis. The integrated FLuc luminescence intensity within the tumour region was quantified and subsequently normalized based on the initial measurement of the same tumour, resulting in the normalized tumour size for monitoring tumour growth.

### In vivo tumour models

To evaluate the efficacy of combinatorial immunotherapy, NSG mice with bilateral subcutaneous tumours were established by injecting 0.5 million PSMA^+^/FLuc^+^ PC-3-CaDox cells with inducible tCD19 into both hind limbs. On day 10, mice were split into two groups: one group received 1 mg kg^−1^ doxycycline (i.p.) to induce the CaDox system, and the control group received no doxycycline. FUS stimulation was applied to the left-side tumours in all mice. Subsequently, synNotch CAR T cells were injected into both the left-side and right-side tumours in all mice. Tumour growth was monitored using BLI from day 5, with measurements taken every 3 to 4 days until mice reached the humane end-point.

In a separate study to evaluate the in vivo delivery and activation of the CaDox system via AAV vectors, NSG mice were inoculated with PSMA^+^/FLuc^+^ PC-3 cells without prior genetic engineering to establish bilateral subcutaneous tumours. The CaDox AAVs were produced by the GT3 Core Facility of the Salk Institute with a titre of 1.34 × 10^12^ genome copy (GC) per millilitre (AAV2-TRE3G-tCD19, CaDox reporter) and 1.35 × 10^12^ GC ml^−1^ (AAV2-PGK-rTetR-tNFAT-VP64, CaDox regulator). On day 10, the AAV viruses were mixed at a 1:1 ratio (9 μl each) and injected subcutaneously near the tumours to facilitate localized gene delivery. Following the established protocol, on day 13, half of the mice received doxycycline (1 mg kg^−1^, i.p.), while FUS stimulation was applied to the left-side tumours across all mice, as previously described. This was immediately followed by the administration of synNotch CAR T cells into both tumours. Tumour growth was monitored using BLI from day 5, and tumour volume (*V*) was measured by caliper from day 9 (*V* = 0.5 × *L* × *W*^2^; *L*, length; *W*, width).

To evaluate the systemic in vivo delivery and activation of the CaDox system, lentiviral vectors carrying the CaDox circuit were injected into NSG mice on day 5, mice that had been implanted with PSMA^+^/FLuc^+^ PC-3 cells without prior genetic engineering. On day 12, one group of mice underwent combined treatment, while another group was left untreated. Following this, synNotch CAR T cells were administrated to both groups. Tumour growth was monitored using IVIS imaging and caliper measurements as previously described.

To assess the efficacy of the systemic delivery of synNotch CAR T cells, NSG mice with bilateral subcutaneous PSMA^+^/FLuc^+^ PC-3-CaDox cells were established. On day 8, synNotch CAR T cells (10 million cells per mouse) were administered intravenously. The animals received doxycycline (10 mg kg^−1^, i.p.) and FUS treatment (applied to the left-side tumours) on days 10, 12 and 14 to activate the CaDox system. Tumour growth was monitored using BLI and caliper measurements, following the same protocol as previously described.

### Reporting summary

Further information on research design is available in the Nature Portfolio Reporting Summary linked to this article.

## Online content

Any methods, additional references, Nature Portfolio reporting summaries, source data, extended data, supplementary information, acknowledgements, peer review information; details of author contributions and competing interests; and statements of data and code availability are available at 10.1038/s41563-025-02391-8.

## Supplementary information

Supplementary Information Supplementary Figs. 1–21, Tables 1 and 2, Discussion, Methods and Note.

Reporting Summary

Supplementary Data 1 Statistical source data.

## Source data

Source Data Fig. 1 Statistical source data.

Source Data Fig. 2 Statistical source data.

Source Data Fig. 3 Statistical source data.

Source Data Fig. 4 Statistical source data.

Source Data Fig. 5 Statistical source data.

Source Data Fig. 6 Statistical source data.

## Data Availability

All data generated or analysed during this study are included in Supplementary Information. Source data are provided with this paper.

## Code availability

The code repository for the proportional–integral–derivative (PID) controller of the in-house-built FUS system has been published in our previous study^2^. The code for image analysis and quantification used in this study is available via Zenodo at 10.5281/zenodo.15734163 (ref. ^50^) and is licensed under the MIT license.
