# Supplementary material for: Tumour priming by ultrasound mechanogenetics for CAR T therapy
Source: Nat Mater. 2025 Oct 31;25(2):310–21. doi: 10.1038/s41563-025-02391-8 (PMC12867766; doi:10.1038/s41563-025-02391-8)
Supplement: Supplementary file 1 — Supplementary Figs. 1–21, Tables 1 and 2, Discussion, Methods and Note. [file 41563_2025_2391_MOESM1_ESM.pdf]

nature materials

Supplementary information https://doi.org/10.1038/s41563-025-02391-8

Tumour priming by ultrasound mechanogenetics for CAR T therapy

In the format provided by the authors and unedited

Table of Contents

Supplementary Methods 2
Supplementary Note 6
Supplementary Figures and Figure Legends 10
Supplementary Tables 33
Supplementary References 35

Supplementary Methods

This section contains detailed in vitro experimental procedures, including protocols for CRISPR-mediated gene knockout, calcium imaging, temperature sensing, luciferase and fluorescence-based assays, protein detection, 3D cell culture, and viral vector biodistribution detection.

Mechanistic study of FUS-induced calcium responses

To study the mechanism of FUS-induced calcium responses in cancer cells, we utilized CRISPR RNP-mediated knockout, with sgRNAs designed via synthego's CRISPR Design Tool (www.synthego.com/products/bioinformatics/crispr-design-tool) to knockout PANX1 (UGACGCCAGGAGAAAGAACU). For the knockout procedure, sgRNA and Cas9 proteins were mixed in a 3:1 molar ratio to produce RNP particles, involving cells prepared at 0.2 million per reaction. Electroporation was then performed using the 4D Nucleofector® (Lonza, X unit), adhering to cell-type specific settings recommended by the manufacturer. After the electroporated cells grow into 1 million cells, genomic DNA was extracted using a *Quick*-DNA Miniprep kit (Zymo Research, D3024) and CRISPR target regions were amplified by PCR and sent out for sanger sequencing (Genewiz). The KO efficiency was assessed using ICE CRISPR Analysis Tool (www.synthego.com/products/bioinformatics/crispr-analysis).

Live-cell imaging and quantification

Live-cell calcium imaging was performed to monitor intracellular calcium dynamics in response to FUS stimulation. Cells were loaded with the calcium-sensitive fluorescent dye, Fluo-4 AM (Thermo Fisher Scientific, F14201), following the manufacturer's instructions. Briefly, cells were incubated with 5 µM Fluo-4 AM and 0.02% Pluronic F-127 (Thermo Fisher Scientific, P3000MP) in a buffer solution for 30 minutes at 37°C. After incubation, cells were washed in fresh buffer solution to remove excess dye. The loaded cells were then placed on an inverted epifluorescence microscope (Nikon, Eclipse Ti) for imaging, and MetaMorph 7.8 software (Molecular Devices) was used to control image acquisition and time-lapse settings. Alternatively, cells were stably transduced with a genetically encoded calcium sensor, R-Geco1[6], to monitor intracellular calcium dynamics in organoids. R-GECO1 was introduced into cells using lentiviral infection, followed by FACS sorting to generate stable cell lines expressing the calcium sensor. Once stable cell lines were established, they were used to generate organoids. Time-lapse imaging was performed to capture the temporal changes in intracellular calcium levels, and the acquired data were analyzed either using ImageJ software (National Institutes of Health) or using CellProfiler 4.2.6 (Broad Institute)[7] with custom-written pipelines to segment cells and calculate normalized changes in fluorescence ($\Delta F/F$). To quantify cellular responses to FUS stimulation, the Cell Response Index (CRI) was calculated for each timelapse image using custom code implemented in Matlab R2023b (MathWorks). CRI reflects both the magnitude of fluorescence changes and the proportion of responding cells. $\Delta F/F$ for each cell was calculated, adjusting for pre-stimulation levels. Only cells exceeding a predefined RMS noise threshold were considered responsive. CRI is the product of the mean maximal delta F/F among these cells and their response rate, serving as an aggregate measure of response.

To assess temperature changes in spheroids upon FUS stimulation, a mCherry-based fluorescence thermosensing method[8] was employed in U-87MG spheroids stably expressing mCherry. For temperature calibration, a control experiment was conducted using a stage temperature controller (Instec, mK2000) to heat spheroids from 37°C to 45°C, while recording mCherry fluorescence

intensity under controlled conditions. mCherry intensity was normalized for each trial, with the initial intensity at 37°C set to 100%, and the percentage decrease in fluorescence was measured relative to actual temperature changes recorded by the stage controller. The average percentage change in mCherry intensity per degree Celsius was determined and used as a conversion factor to estimate temperature shifts in FUS-treated spheroids. This calibration curve enabled quantitative conversion of mCherry fluorescence changes into temperature values, allowing for a direct comparison of thermal effects between FUS-treated and non-FUS-treated spheroids.

***In vitro* luciferase-based assay**

For the luciferase-based assay, the Dual-Glo® Luciferase Assay System (Promega, E2920) was used to measure the activity of luciferase in the cell samples according to the manufacturer's instructions. Briefly, engineered HEK293T cells (0.05M) were seeded in a 96-well plate and allowed to adhere overnight. The next day, the cells were treated with various experimental conditions. At the end of the treatment period, the cells were washed with PBS 3 times and replenished with media for incubation before the assay. After 6 hours, the Dual-Glo Luciferase Reagent was added to each well and incubated at room temperature for 3 minutes to lyse the cells and stabilize the luminescent signal. The luminescence from firefly luciferase (Fluc) was measured using a plate reader (Tecan, Infinite M200 Pro). Afterward, the Dual-Glo Stop & Glo Reagent was added to each well, and the plate was incubated for another 3 minutes at room temperature to quench the Fluc signal and generate luminescence from Renilla luciferase (Rluc). The Rluc luminescence was then measured using the same plate reader. The Rluc (constitutive) luminescence values were used to normalize the Fluc (inducible) data, accounting for any variability or cell number, providing a more accurate representation of the experimental effects on luciferase activity.

For the extracellular ATP measurement, we used the CellTiter-Glo® 2.0 cell viability assay (Promega, G9241) according to the manufacturer's instructions. PC-3 cells on imaging dishes were stimulated with FUS for 30 minutes, applying a pattern of 1 minute every 5 minutes. After stimulation, the culture media were mixed with an equal volume of CellTiter-Glo® 2.0 reagent and incubated at room temperature for 3 minutes to stabilize the luminescent signal. The luminescence was subsequently measured using a plate reader. The data obtained from this assay offered insights into extracellular ATP levels in the presence and absence of FUS stimulation.

***In vitro* fluorescence-based assay**

For the fluorescence-based assay, mCherry expression was used as a readout to assess the inducibility of the CaDox system against various treatment conditions. Engineered cancer cells expressing the mCherry gene under the control of the CaDox system were seeded in imaging dishes and allowed to adhere overnight. The following day, the cells were exposed to different experimental conditions to test the CaDox system's response. After the treatment period, the cells were washed three times with PBS and replenished with fresh media before incubation. After 6 hours of incubation, the mCherry fluorescence intensity was measured using an inverted epi-fluorescence microscope with a 20x objective. Images were captured and then analyzed using a custom Python code to segment the cells and calculate the sum of intensities from the segmented regions. This approach allowed for the quantification of mCherry expression levels, providing a reliable measure of the CaDox system's inducibility under the various treatment conditions.

Inducible protein detection

To assess the expression of inducible tCD19 in PC-3 cells, we utilized a flow cytometry approach. After induction and harvesting, cells were incubated with 5 µL of anti-CD19 (clone HIB19) monoclonal antibody conjugated with APC (BioLegend, 302218, dilution 1:20) per million cells at 4°C for 30 minutes. Following incubation, cells were washed three times with DPBS and analyzed using a BD Accuri C6 Plus flow analyzer. Additionally, immunofluorescence microscopy was employed to further validate the expression. Induced cells were fixed with Fixation Buffer (BioLegend, 420801), blocked using standard protocols, and then stained with an anti-CD19 (clone HIB19) monoclonal antibody conjugated with AF647 (BioLegend, 302220) at a 1:100 dilution, and Hoechst 33342 (Invitrogen, R37165) for nuclear visualization. After washing, samples were mounted using mounting medium (Vector Laboratories, H-1900) and examined under a fluorescence microscope. This approach enabled precise detection and localization of inducible tCD19 expression within the cells.

To assess the expression of inducible PSMA-CAR in synNotch CAR T cells, we followed the same protocol for flow cytometry, using an anti-c-Myc antibody conjugated with AF647 (BioLegend, 626809, dilution 1:20) to detect the c-Myc tag inserted into the extracellular domain of the PSMA-CAR protein.

3D cell culture

To generate PC-3 cell-derived 3D organoids, 50,000 prostate cancer PC-3 cells were suspended with 10 µL advanced DMEM/F12 media (Life Technologies, 12634010) supplemented with 10 mM HEPES (Life Technologies, 1560106), 2 mM Glutamax, and 1% P/S[9]. The cell mixture was gently mixed with 40 µL of Matrigel HC (Corning, CB354248) on ice. The resulting mixture was then deposited at the center of a glass-bottom dish and allowed to polymerize at room temperature for 20 minutes. Following the polymerization, 2 mL of complete organoid culture media (composition provided in Supplementary Table 2), supplemented with 10 µL of Y-27632 dihydrochloride (ROCK inhibitor), was added to the dish. Dishes were incubated in a 37°C humidified incubator with 5% CO_2, placed upside down to enhance organoid formation. The culture medium was refreshed every 3-4 days. After 5-7 days, medium without Y-27632 was used to maintain the cultures. Experiments were conducted using mature organoids cultured for 12-15 days.

Spheroids from the breast cancer cell line MDA-MB-231 and the glioblastoma cell line U87-MG were generated using a previously reported magnetic-field-assisted assembly technique[10]. Briefly, 10,000 cells were seeded into each well of a 96-well plate containing culture media supplemented with Diethylenetriaminepentaacetic acid gadolinium (III) dihydrogen salt hydrate (Gd-DTPA, Sigma Aldrich, 381667). The plate was then placed over quartets of square cuboid magnets and cultured in a CO_2 incubator for 3-6 hours to facilitate cell aggregate formation. After magnet removal, the aggregates were cultured for an additional 48 hours. For imaging, the spheroid bodies formed in the 96-well plates were transferred to glass-bottom dishes. The spheroids were then embedded in a 4 mg/mL collagen gel (Corning, CB354249) supplemented with 10 µg/mL fibronectin (Sigma Aldrich, F1141) and imaged after 48 hours.

Biodistribution analysis of viral vector-mediated *in vivo* circuit delivery

To evaluate the *in vivo* delivery and biodistribution of CaDox circuit, two different injection methods were used: systemic intraperitoneal (i.p.) injection and focal injection. For systematic delivery, mice were injected i.p. with AAV2-CMV-Fluc at a dose of 4E11 GC per injection in 100

μL of sterile PBS. BLI was performed at multiple time points (Days 1, 3, 5, and 7 post-injection) to assess the systemic distribution of the viral vector. Thirty days post-injection, mice were euthanized, and various organs (heart, liver, lung, spleen, kidneys, bladder, and testis) were harvested for ex vivo BLI to confirm the presence of the viral vector.

For focal delivery, AAV2-CMV-Fluc was injected subcutaneously next to the tumor site at a dose of 2E10 GC per injection in 20 μL of sterile PBS. *In vivo* BLI was conducted at different time points (Pre, Days 1, 3, 5, 7, and 30) to monitor the localization and potential spread of the viral vector beyond the tumor site. On Day 30, mice were euthanized, and the tumours and adjacent tissues were excised and subjected to ex vivo BLI to evaluate viral leakage beyond the tumor.

Supplementary Note

A variety of techniques have been developed to perturb cells and tissues through energy-based modalities, such as optics[1], magnetics[2], and ultrasound[3]. Among the listed modalities, optogenetics has emerged as a particularly successful approach, combining molecular sensors of lights and inducible gene expression systems to control protein production and cellular functions[4-9]. Optogenetics involves the use of light-sensitive proteins to manipulate cellular activity in response to specific wavelengths of light[1]. This powerful tool has enabled researchers to control various aspects of cellular function with unprecedented precision, both spatially and temporally. The versatility of optogenetics has led to its widespread adoption across various disciplines, including neuroscience[1, 9, 10], immunology[11-13], and developmental biology[14], among others. However, a major challenge in translating optogenetics to clinical applications is the limited penetration depth of light, which restricts its utility in deeper tissues and organs.

FUS-based therapy has already been used in clinics to treat prostate cancer, the second most common cancer in men in the United States. Standard treatments for localized high-risk prostate cancer, such as radical prostatectomy or radiation, are effective but often lead to complications like urinary incontinence, erectile dysfunction, or bowel urgency[15] which impair patients' quality of life[16, 17]. Even with non-invasive monitoring of low-grade cancer, the risk of undetected progression persists, prompting many to choose radical treatments. Focal therapies like cryoablation and high-intensity focused ultrasonography aim to reduce overtreatment[18, 19] yet they still pose risks of collateral damage to benign tissues and neurovascular structures, leading to urogenital dysfunction[18]. Additionally, around 30% of patients experience clinically significant prostate cancer recurrence within two years[18]. Hence, there is a critical need for advanced focal treatments that effectively manage prostate cancer while preserving patients' quality of life.

The mechano-sensitivity of cancer cells has recently emerged as a critical character[1-3]. These cells can sense and respond to the physical properties of their surrounding extracellular matrix (ECM), such as stiffness, topography, and composition[4-6]. Interestingly, several studies have reported that some cancer cells are sensitive to FUS stimulation, resulting in calcium responses[7, 8], suggesting that this modality could be harnessed to target and manipulate the genetics of these tumor cells in preparation for subsequent therapy. However, to fully exploit this potential, there is a need to translate FUS-induced calcium signals into user-defined gene activations for the precise control of cellular functions.

FUS is a non-invasive technique that generates mechanical energy in the form of ultrasound waves. At the tissue level, FUS can non-invasively open the blood-brain-barrier for targeted drug delivery[9], induce temporary cellular permeability via sonoporation to enhance drug or gene uptake[10], and facilitate non-thermal tissue destruction through histotripsy[11]. On a cellular scale, when these waves interact with cells, they can cause mechanical stimulation, which in turn can trigger various mechanotransduction pathways including integrin signaling[12], the FAK/MAPK/ERK signaling cascade[13, 14], and the activation of mechanosensitive ion channels like Piezo1[15, 16], MscL[17, 18], or TRPA1[19]. The activation of these ion channels by FUS has been explored for its potential to affect cellular responses. However, thus far, the use of ultrasound to stimulate cells has been primarily limited to modulating endogenous signaling and gene expression, which typically lacks the specificity of genetic and cellular controls. We demonstrate the feasibility of FUS-induced mechanical stimulation to directly control user-designed gene expression and cellular functions without the need of a cofactor such as microbubbles. Our study hence represents a new

advancement in the field of sonogenetics by demonstrating the versatility and adaptability of FUS-based mechanogenetics, expanding toolkits of sonogenetics alongside previously established FUS-based thermogenetics. This could potentially pave the way for more diverse and multiplex applications of ultrasound in specific gene regulation and cellular control.

The present system relies on the inherent mechanosensitivity of cancer cells, specifically on mechanosensitive ATP release and corresponding calcium responses in prostate cancer PC-3 cells[20]. The activation of mechanosensitive hemichannels leads to ATP release, which in turn stimulates P_2Y purinergic receptors. This stimulation results in an increase in inositol 1,4,5-triphosphate (IP_3) levels and the subsequent release of calcium, further activating ATP release[21] , thereby establishing a positive feedback loop. Our findings are consistent with the existing understanding of the role of hemichannel such as PANX1 in initiating and propagating intercellular calcium waves (see supplementary Fig. 2). FUS-induced ATP release can thus facilitate the conversion of localized stimulation by FUS into synchronized response from the whole targeted tissue masses, such as solid tumours.

In the current study, we primarily focused on prostate cancer, using PC-3 cells as our model for FUS-mediated mechanogenetics. However, we also demonstrated that FUS-mediated calcium responses are conserved in various cancer cell types, including breast cancer cells and glioblastoma. The modular design of our FUS-CaDox system can hence be readily extendable for tissue priming of other cancer types. Indeed, the functionality of CaDox system can be observed in different cell types tested, including Jurkat T cells and MDA-MB-231 human breast cancer cells, highlighting its broad applicability (Supplementary Fig. 7). Some studies have suggested potential roles for PANX1 in cancer progression; for example, metastatic cell survival during microvascular deformation is facilitated by increased ATP release from mechanosensitive PANX1 channels activated by membrane stretch, which then acts as an autocrine suppressor of deformation-induced apoptosis through P_2Y-purinergic receptors[22]. In addition, different cancer cell types may rely on distinct mechanotransduction pathways, shaped by their mechanosensitive channel composition, expression levels, and intrinsic mechanical properties such as cellular stiffness, which warrants further investigation[6, 23-25]. Although further investigation is required to gain a mechanistic understanding of the relationship between FUS sensitivity and cancer cell properties, the observation that cancer cells tend to respond to FUS more than normal healthy cells suggests a potentially additional therapeutic advantage for this technique, differentiating cancer cells from those normal ones.

To harness the FUS-mediated calcium response for customized transcriptional activities, we developed an AND logic gate circuit termed CaDox, which relies on both calcium and doxycycline for its activation. CaDox is designed to minimize crosstalk between the synthetic circuit and endogenous signaling pathways by employing a truncated NFAT motif and the Tet-On component. While doxycycline caused some minor genetic leakages in the absence of stimulated calcium *in vitro*, the CaDox circuit is highly effective and only responds significantly in the presence of both FUS-induced calcium and doxycycline (Figs. 2G-J). In our *in vivo* killing assay, the doxycycline-only group exhibited no tumor suppression, while the doxycycline and FUS combined group demonstrated significant suppression (Fig. 6 and Supplementary Fig. 17). This suggests that the minor leakage of doxycycline of CaDox may not affect practical applications *in vivo*.

We explored the potential of our mechanogenetic system for immunotherapy by rewiring the circuit to express tCD19 antigen upon FUS activation for tumor priming, enabling precise targeting

by synNotch CAR T cells. This approach offers a clear advantage over conventional cytotoxic/suicide gene therapy for cancer treatment, which not only rely solely on delivery efficiency, which is often suboptimal[26], but also lack spatial and temporal control over gene expression post-administration, leading to significant safety concerns. In contrast, our combinatorial immunotherapy strategy utilizes FUS as an external modulator, allowing the spatiotemporal controls. Additionally, this approach utilizes a subset of infected and induced cancer cells to serve as local “training centers”, activating synNotch CAR T cells to recognize and attack the entire cancer cell population around the tumor region. Furthermore, the CAR expression on the synNotch CAR T cells activated by the local “training centers” at the tumor site is transient in nature and will hence gradually decay when the synNotch CAR T cells stride away from the priming site[27]. Previously, we demonstrated that transiently and locally induced CAR expression significantly reduces off-tumor toxicity, in contrast to constitutively active CAR T cells, which have shown considerable off-tumor effects even with local intratumoral injections[28]. This supports the enhanced safety profile of our 'training center' strategy, where synNotch CAR T cells are activated transiently and locally, minimizing off-tumor toxicity. This will hence allow the high-dosage use of highly potent but less specific CAR without the off-tumor toxicity yet achieving a high treatment efficiency. Although the use of tCD19 as an initiator for synNotch CAR T cells might cause non-selective killing of B cells expressing CD19 (i.e. B cell aplasia), lymphodepletion—a well-established process in CAR T-cell therapy—minimizes the number of B cells in the patient's body to create a time window for treatment[29]. In fact, in clinical trials, fludarabine and cyclophosphamide have been established to pretreat patients for lymphodepletion before CAR T application[30]. B cell aplasia itself is also clinically manageable with immunoglobulin replacement[31, 32].

Looking ahead, it is also important to situate our novel combinatorial immunotherapy approach within the landscape of existing non- or minimally invasive therapies. These include radiopharmaceuticals[55], proton therapy[56], boron neutron capture therapy[57], and tumor treating fields (TTFs)[58], each of which has its own strengths and limitations. Radiopharmaceuticals offer systemic targeting of tumours but can cause unintended radiation exposure to healthy tissues. Proton therapy and BNCT deliver highly precise irradiation but require specialized, high-cost infrastructure. TTFs provide a non-invasive treatment option and effectively target rapidly dividing tumor cells. However, they require long daily usage (≥18 hours/day)[59] and may be less effective against slow-dividing cancer cell populations, particularly in consolidation therapy. Furthermore, the locally trained and activated CAR T cells in our approach can migrate and eliminate nearby tumor cells scattered outside the focal area[60], resulting in efficient and safe eradication of cancer cells in the local tumor site.

While our study demonstrates the potential of the FUS-CaDox system, there are areas that warrant further exploration. First, expanding beyond the use of the PC-3 cell line to incorporate more complex and clinically relevant tumor models could better reflect the diversity of tumor microenvironments. Second, future studies should employ more immunocompetent tumor models to better simulate clinical conditions and allow for the study of systemic immune responses, such as cytokine release syndrome. This will provide a more comprehensive evaluation of the therapy’s effects in the context of a fully functional immune system. Third, although significant tumor suppression was observed, achieving complete eradication remains a key future goal. Optimizing the delivery strategies and treatment protocols, particularly for systemic administration, will be critical to enhancing therapeutic efficacy. These potential investigations may offer exciting

opportunities in the future for advancing the FUS-CaDox system towards more robust and effective cancer treatments. Given the rapid advancements in the field of immunotherapy and the potential impact of our mechanogenetic system, there is a promising horizon for further refinement and integration of this approach into a broader array of clinical applications, paving the way for safer and more effective cancer treatments in the future.

Supplementary Figures and Figure Legends

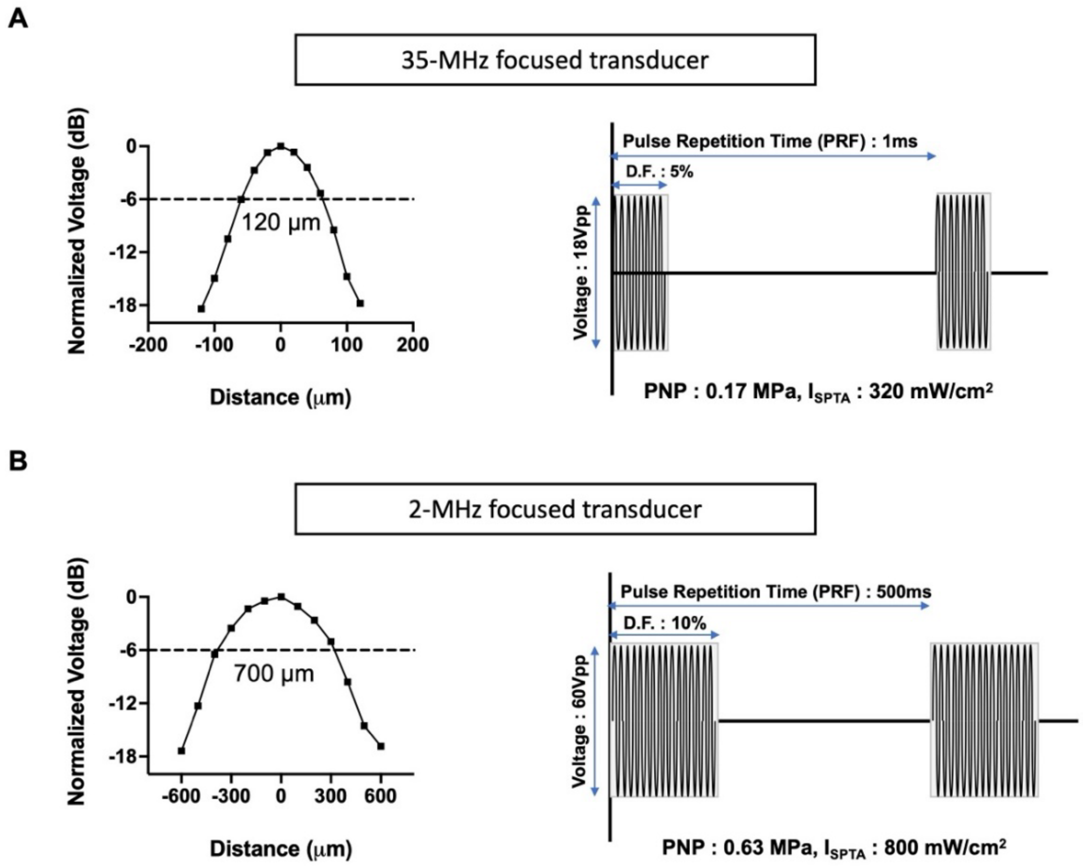

Supplementary Figure 1: Characterization of FUS transducers for *in vitro* studies

Beam profile plots at the focal plan, input parameters, pulsed signal patterns, and output powers for **(A)** 35-MHz single element lithium niobate ultrasound transducer and **(B)** 2-MHz single element PZT ultrasound transducer. Beam profiles and output powers, represented as peak-negative-pressure (PNP) and spatial-peak-temporal-average intensity (I_{SPTA}), were measured using a hydrophone. Note that both transducers were operated within the range of low-intensity pulsed ultrasound (LIPU) scheme.

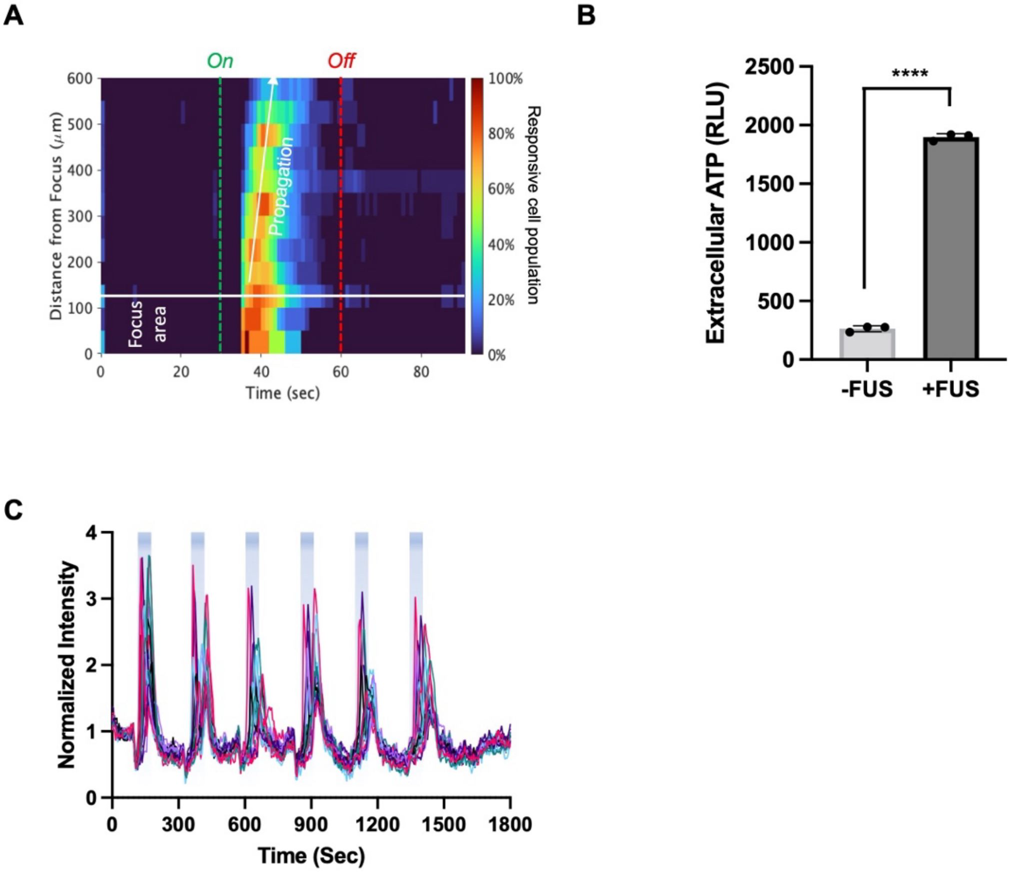

Supplementary Figure 2: FUS-induced calcium responses and repeated stimulation effects

(A) Bivariate histogram showing FUS-induced calcium responses over time, with respect to the distance from the focal point of the transducer. The proportion of responsive cells is depicted in relation to the FUS focus, with green and red dotted lines indicating the timing of FUS stimulation being turned on and off, respectively. Calcium responses were observed beyond the FUS focal area, propagating to distances exceeding 500 µm. **(B)** Quantification of extracellular ATP levels in the imaging media measured by luciferase assay, with or without focused ultrasound (FUS) exposure. Data are presented as mean ± SEM (n=3). Statistical significance was determined using Welch's t-test. ****P < 0.0001. **(C)** Calcium responses traces from multiple short, pulsed FUS stimulations (1 min on, followed by 4 min off, repeated six times). Each trace represents individual cell responses (n=23) at the focus, demonstrating consistent calcium signaling patterns with repeated FUS exposure.

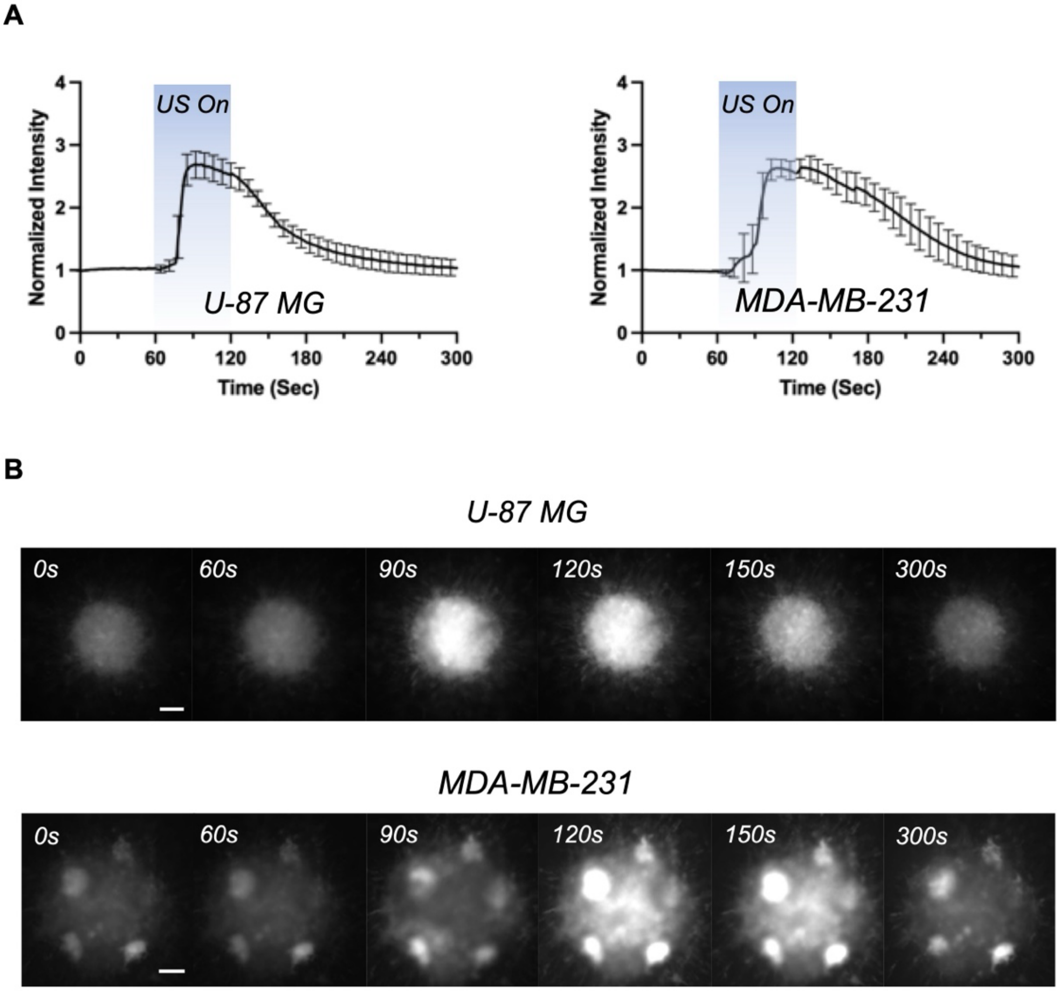

Supplementary Figure 3: FUS induced calcium responses in human breast MDA-MB-231 cells and human glioblastoma U-87 MG cells

(A) Normalized fluorescence intensity over time showing calcium response to FUS stimulation in U-87 MG (left) and MDA-MB-231 (right) cell lines. The 'US On' annotation indicates the period during which FUS stimulation was applied (60s – 120s). Error bars represent standard deviation from the mean (n=8 for each plot). **(B)** Sequential fluorescence images captured at various time points (0s, 60s, 90s, 120s, 150s, 300s) in U-87 MG (top row) and MDA-MB-231 (bottom row) cells, illustrating the dynamic calcium flux within the cell populations. The images are representative of 8 independent experiments. Scale bars: 100 µm.

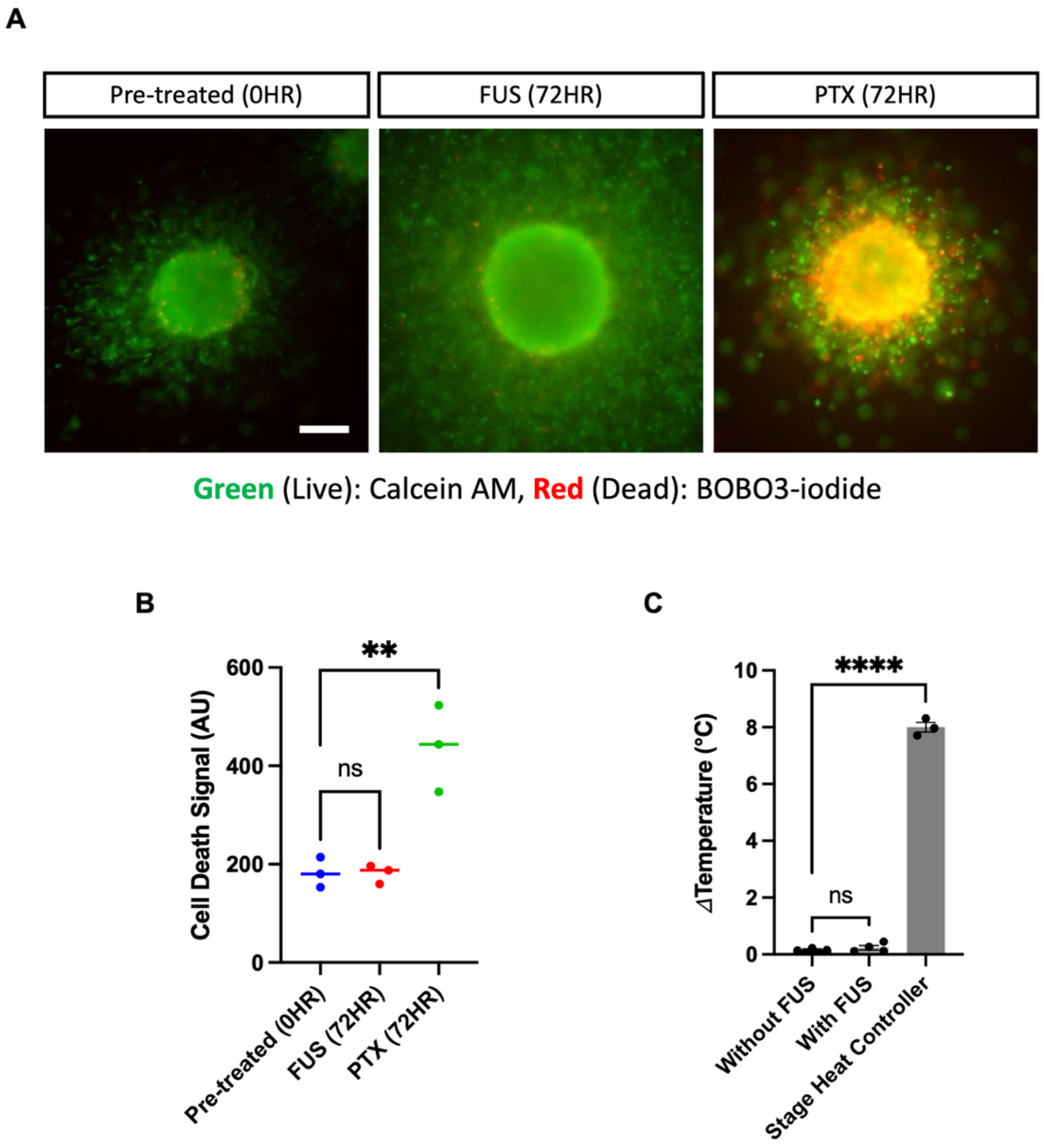

Supplementary Figure 4. FUS stimulation preserves spheroid viability and does not induce significant heating

(A) Representative images of U87 spheroids labeled with Calcein AM (green, live) and BOBO3-iodide (red, dead). Images are shown prior to treatment (Pre-treated, 0 hr), and at 72 hr following either FUS and paclitaxel (PTX, 200 nM) exposure. Scale bar, 200 µm. Images are representatives of >3 independent experiments. **(B)** Quantification of total cell-death signal (arbitrary units, AU) for each condition (n=3). One-way ANOVA followed by Dunnett's post hoc test revealed no significant difference between Pre-treated and FUS groups (adjusted P = 0.9995), while the PTX group showed significantly increased cell death compared to Pre-treated (P = 0.0023). **(C)** Temperature change (ΔT) was measured in spheroids treated without FUS (n=3), with FUS (n=4), or exposed to controlled heating at 45 °C via stage heating (n=3). The stage controller was used to

increase temperature by 8°C (set to 45°C from 37°C) to serve as a positive control. One-way ANOVA with Tukey's test revealed no significant difference between Without FUS and FUS groups (adjusted $P = 0.9996$), but significant increases were observed when comparing either group to the Stage Heat Control group (****$P < 0.0001$). Error bars represent standard deviation (SD).

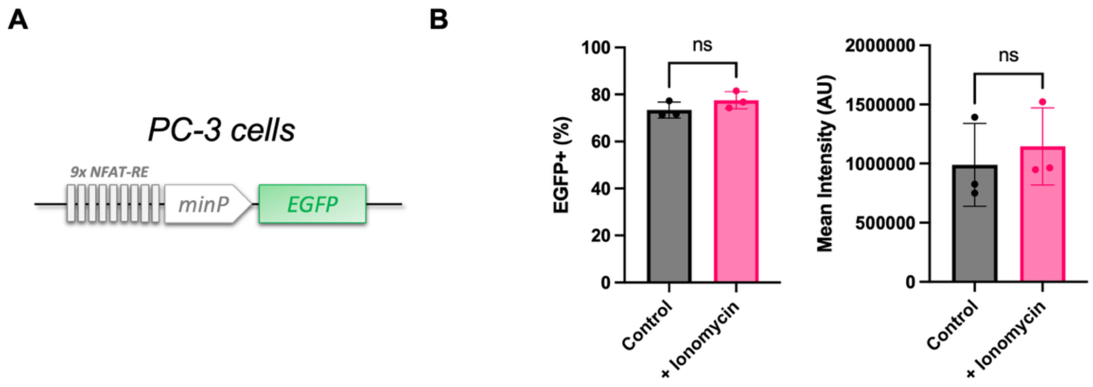

Supplementary Figure 5: Genetic leakages from the NFAT-RE based circuit. (A) The circuit features nine repeats of NFAT response elements (NFAT-RE) followed by a minimal promoter, with EGFP as the inducible reporter gene. **(B)** Characterization of the NFAT-RE-based gene circuit with and without ionomycin (1µM) in PC-3 cells. Data represent mean ± SEM from three biological replicates per group (n = 3 independent wells). Statistical significance was assessed using an unpaired two-tailed Welch's t-test (P = 0.6038; t = 0.5627, df = 3.50). No significant difference was observed.

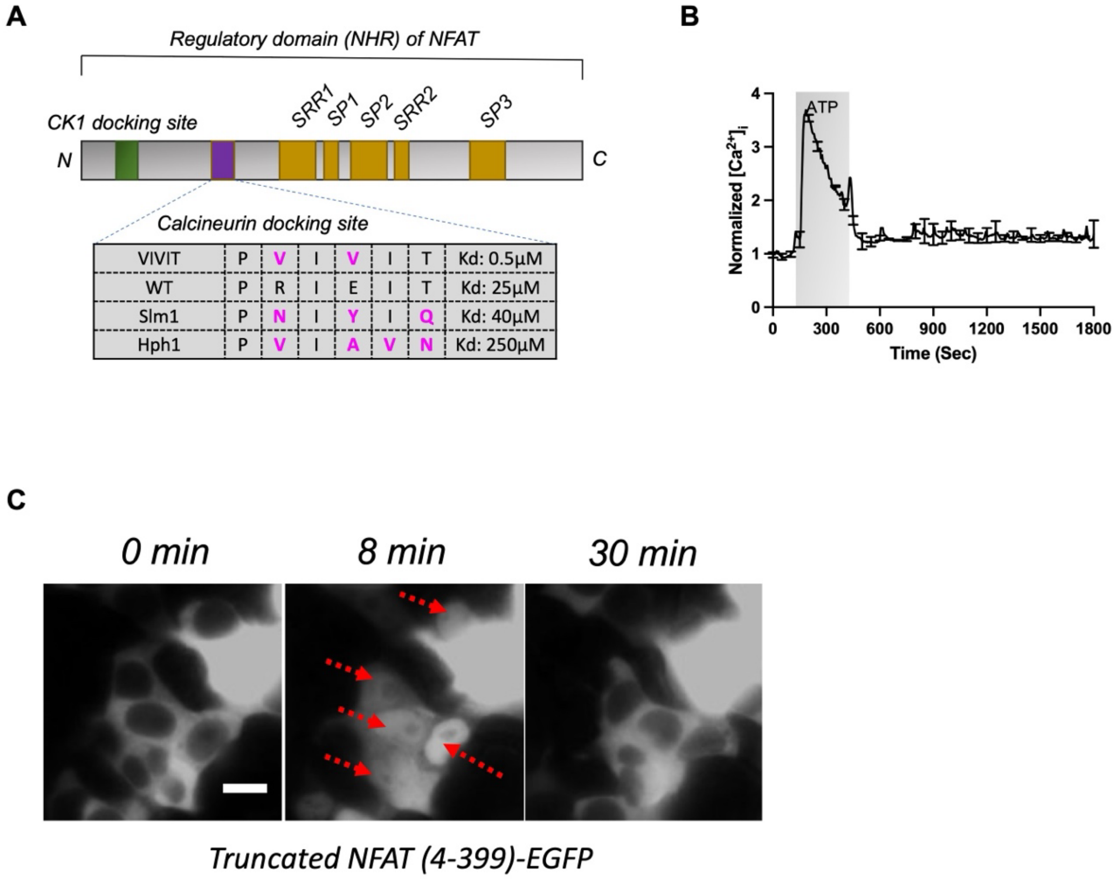

Supplementary Figure 6: Engineering of NFAT mutants with alterations at the calcineurin docking site. (A) Schematic diagram depicting the domain structure of the NFAT protein and a list of mutations at the calcineurin docking site. **(B)** Intracellular calcium response upon incubation with 60 μM ATP. ATP was added at the 2-minute mark and washed off at the 7-minute mark, as indicated by the grey area. n=5. **(C)** Timelapse images of truncated NFAT (4-399)-EGFP upon ATP induction. Red arrows highlight the cells with clear nuclear translocation of truncated NFAT. Images are representative of 5 independent experiments. Error bars represent the standard deviation (SD).

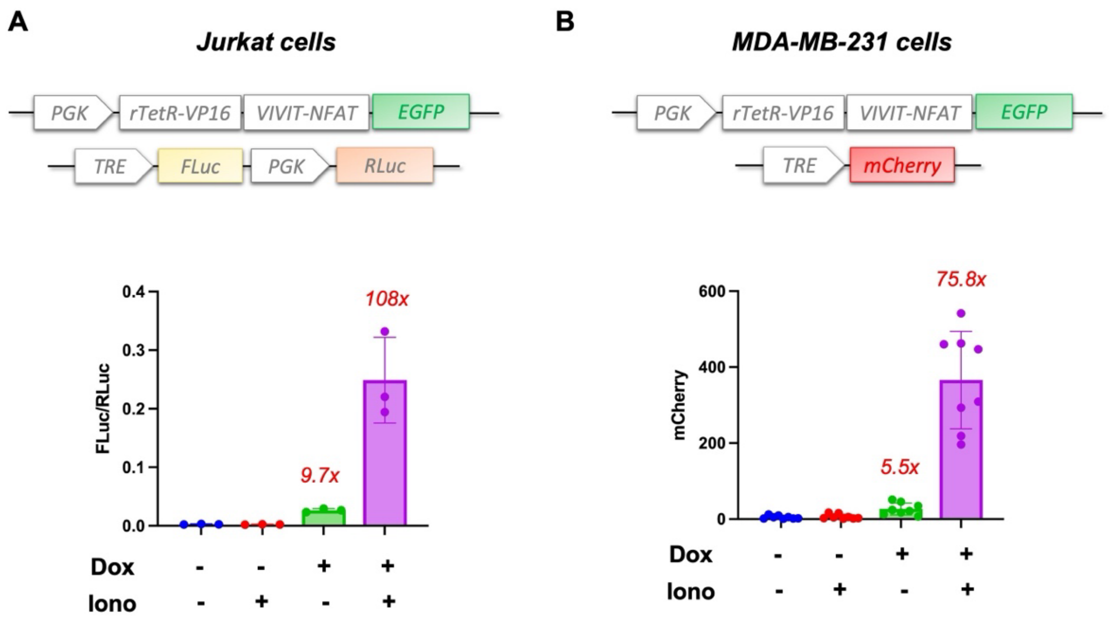

Supplementary Figure 7: The functionality of CaDox system in different cell types. The CaDox system demonstrates consistent functionality across various cell types, including **(A)** Jurkat T cells (n=3) and **(B)** MDA-MB-231 human breast cancer cells (n=8), highlighting its broad applicability. Error bars represent the standard deviation (SD).

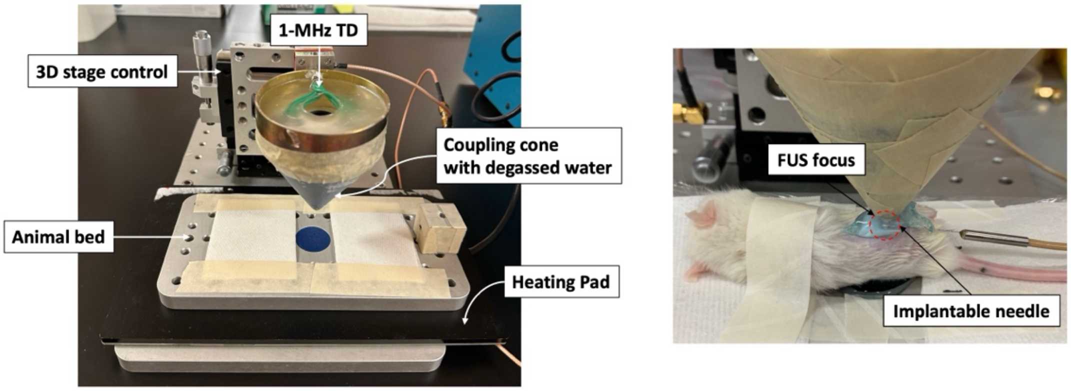

Supplementary Figure 8: The custom FUS stimulation system for animal studies. The image displays the in-house developed FUS stimulation system, including the 1-MHz transducer with a 3D-printed coupling cone designed to hold degassed water along the acoustic path to the animals, the feedback-controlled temperature pad used as the animal bed to maintain body temperature under anesthesia, and the set of manual translational stages employed to control the transducer's location. The coupling cone features a 4 mm diameter opening at the tip, sealed with an acoustically transparent Mylar film to prevent water leakage. Additionally, a close-up image of the animal with acoustic gel and the transducer positioned for FUS stimulation is shown at the right side. A needle-type thermocouple was inserted into the animal's body at the FUS focus region to measure the temperature changes induced by the stimulation.

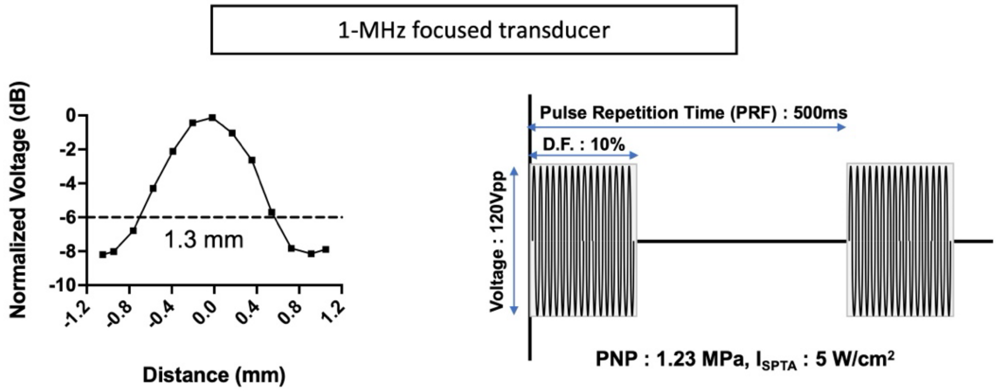

Supplementary Figure 9: Characterization of FUS transducer for *in vivo* study. Beam profile plots, input parameters, pulsed signal patterns, and output powers for 1-MHz single element PZT ultrasound transducer.

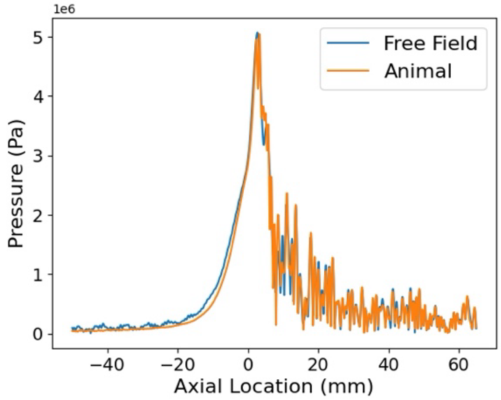

Supplementary Figure 10: Acoustic wave propagation simulation. Comparison of the pressure field for acoustic wave propagation in two different scenarios: free field (water) and within an animal body consisting of skin, muscle, and tumor. All required parameters for the simulation were referenced from relevant literatures (See Methods). The blue line represents the simulation in the free field, while the orange line illustrates the simulation within the animal body, demonstrating the differences in pressure distribution across the axial direction. The maximum pressure in the free field is approximately 5.071 MPa at a location of 2.71 mm, while the maximum pressure in the animal body is approximately 5.043 MPa at a location of 3.33 mm. The difference in maximum pressure is about -27,653 Pa, with a percentage difference of approximately -0.55%.

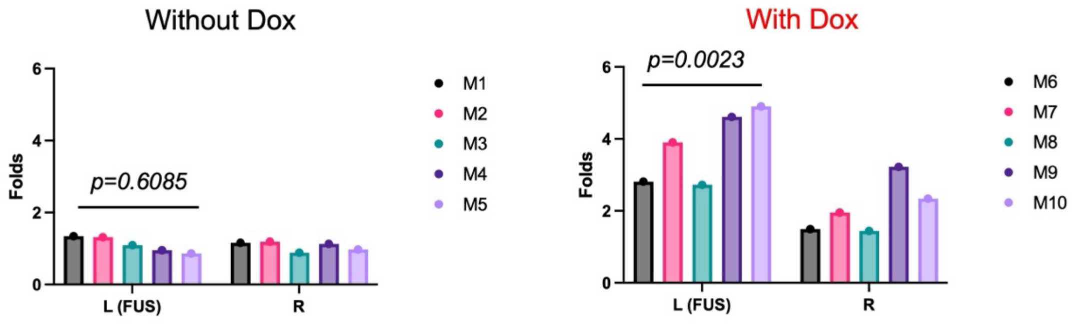

Supplementary Figure 11: *In vivo* gene expression of the CaDox system in individual mice. Mice without doxycycline administration (M1 to M5) did not show a difference between tumors with FUS (left tumors) and without FUS (right tumors). In contrast, mice with doxycycline administration (M6 to M10) exhibited a statistically significant contrast between left and right tumors. The gene activations were measured by BLI imaging at 6 hours from the treatment. This indicates that, consistent with *in vitro* assays, mechanical stimulation by FUS can induce gene expression gated by doxycycline.

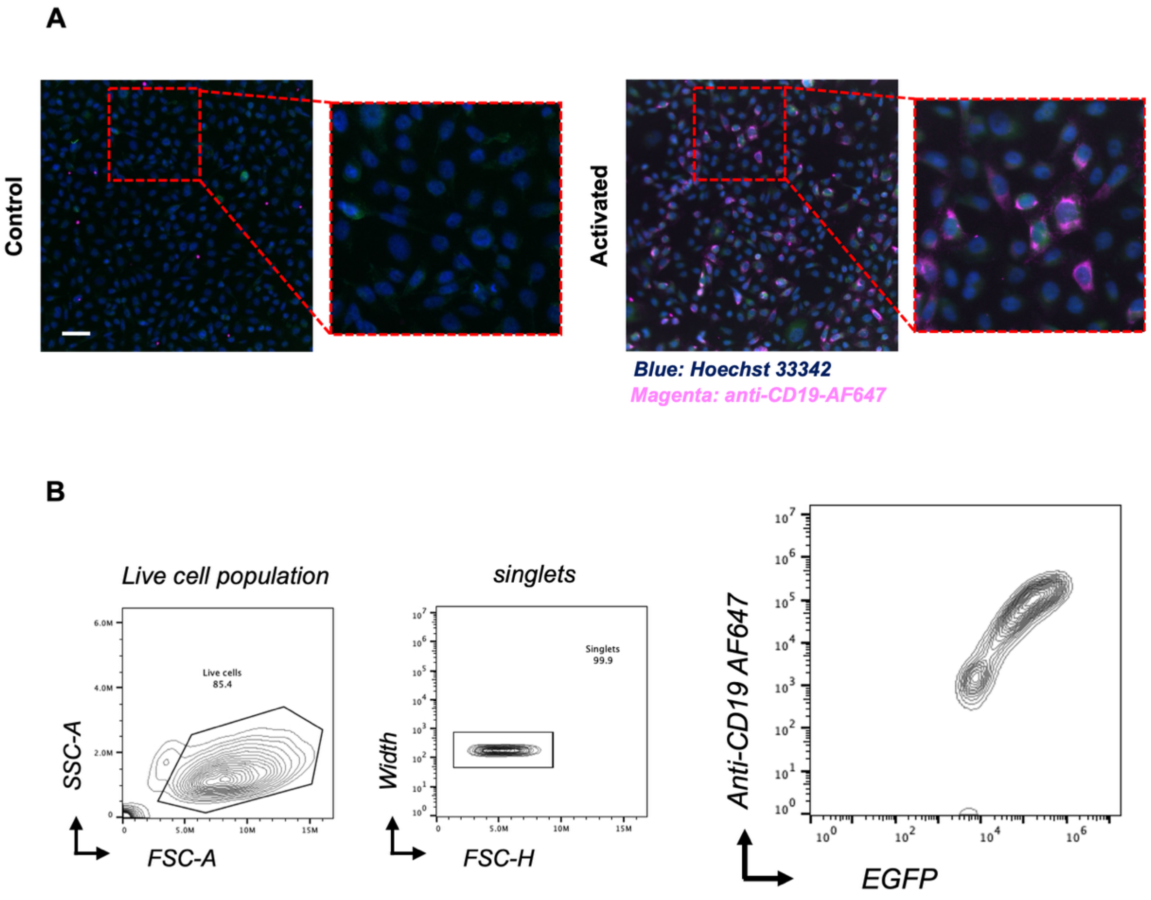

Supplementary Figure 12: Inducible tCD19 expression in PC-3 cells. (A) Immunofluorescence staining of PC-3-CaDox-tCD19 cells. The control sample (top) and activated sample (bottom) demonstrating truncated CD19 expression following induction with doxycycline (200 nM) and ATP (60 µM). Scale bar: 500 µm. **(B)** Flow cytometry analysis of tCD19 in PC-3 cells, plotting EGFP intensity against AF647 intensity from anti-CD19-AF647 staining. EGFP expression corresponds well with CD19 staining, indicating that the induced tCD19 on the plasma membrane is effectively recognized by the anti-CD19 antibody.

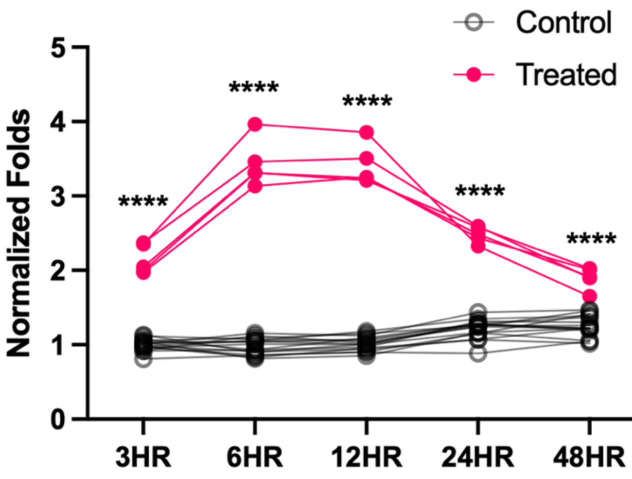

Supplementary Figure 13: Normalized fold changes of tCD19 expression over 48 hours. PC-3-CaDox-tCD19-EGFP cells were subjected to no treatment (NC, n = 13) or combined treatment with doxycycline and focused ultrasound (Dox + FUS, n = 5). Normalized tCD19 expression was quantified at multiple time points. Data are shown as mean ± SEM with individual data points overlaid. Statistical analysis was performed using two-way ANOVA followed by Šidák's multiple comparisons test. **** indicates adjusted $P < 0.0001$.

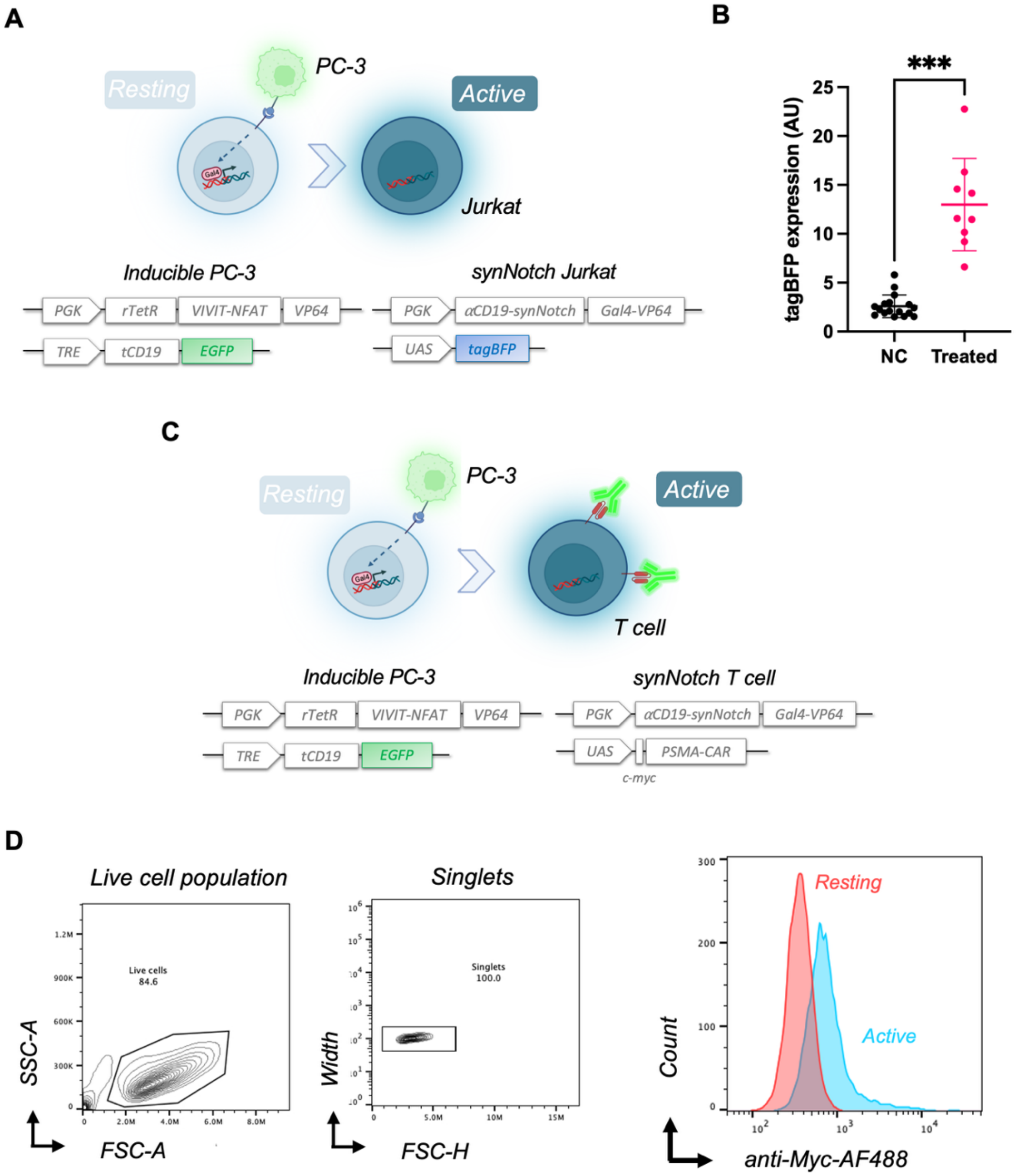

Supplementary Figure 14: Verification of anti-CD19 synNotch activation against inducible tCD19. (A) Schematic illustrating the activation of synNotch Jurkat T cells engineered to express a fluorescent reporter (tagBFP) upon interaction with PC-3 cells expressing inducible tCD19. **(B)** Quantification of tagBFP expression in synNotch Jurkat T cells following activation by tCD19 on PC-3-CaDox-tCD19 cells under different conditions: no treatment (n=17), and combined doxycycline and FUS treatment (n=9). Error bars represent SD. Statistical significance was determined by unpaired Welch's t test. P = 0.0001 (adjusted). **(C)** Schematic illustrating the activation of synNotch T cells engineered to express PSMA CAR upon interaction with PC-3 cells expressing inducible tCD19. **(D)** Flow cytometry analysis showing anti-Myc staining (AF488) in synNotch T cells following activation by tCD19 on PC-3 cells under resting (no treatment) and

active (combined treatment) conditions. Created with BioRender.com. Licensed to Yoon, C. (2025) https://BioRender.com/k64u782.

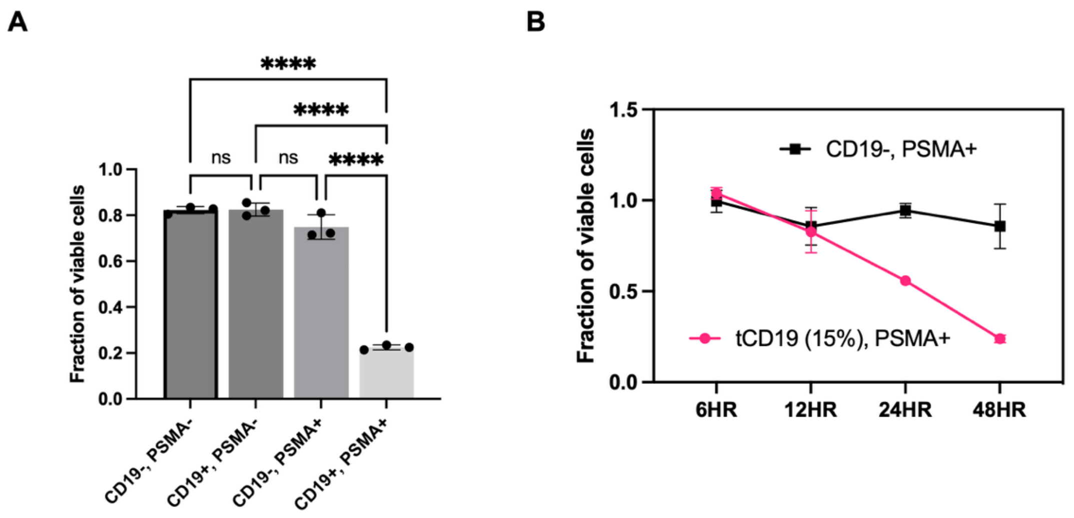

Supplementary Figure 15: SynNotch CAR T cell cytotoxicity against different control groups. (A) Viability of various groups of PC-3 cells—CD19⁻/PSMA⁻, CD19⁺/PSMA⁻, CD19⁻/PSMA⁺, and CD19⁺/PSMA⁺—after co-culture with synNotch CAR T cells for 24 hours at a 1:1 effector-to-target ratio. Error bars represent SD. Statistical analysis was performed using one-way ANOVA with Sidak's multiple comparisons test. Significant differences were observed between the CD19⁺/PSMA⁺ group and all others (adjusted $P < 0.0001$); other comparisons were not significant (adjusted $P > 0.1$). **(B)** Comparison of synNotch CAR-mediated killing of PC-3-PSMA⁺ cells, which show a 15% tCD19 expression from those induced PC-3-CaDox-tCD19 PSMA⁺ cells. This result indicates that a fraction of cells expressing the clinically validated tCD19 can activate synNotch CAR T cells to kill the whole cancer population expressing the common antigen at the tumor site. N=3. Error bars represent SEM.

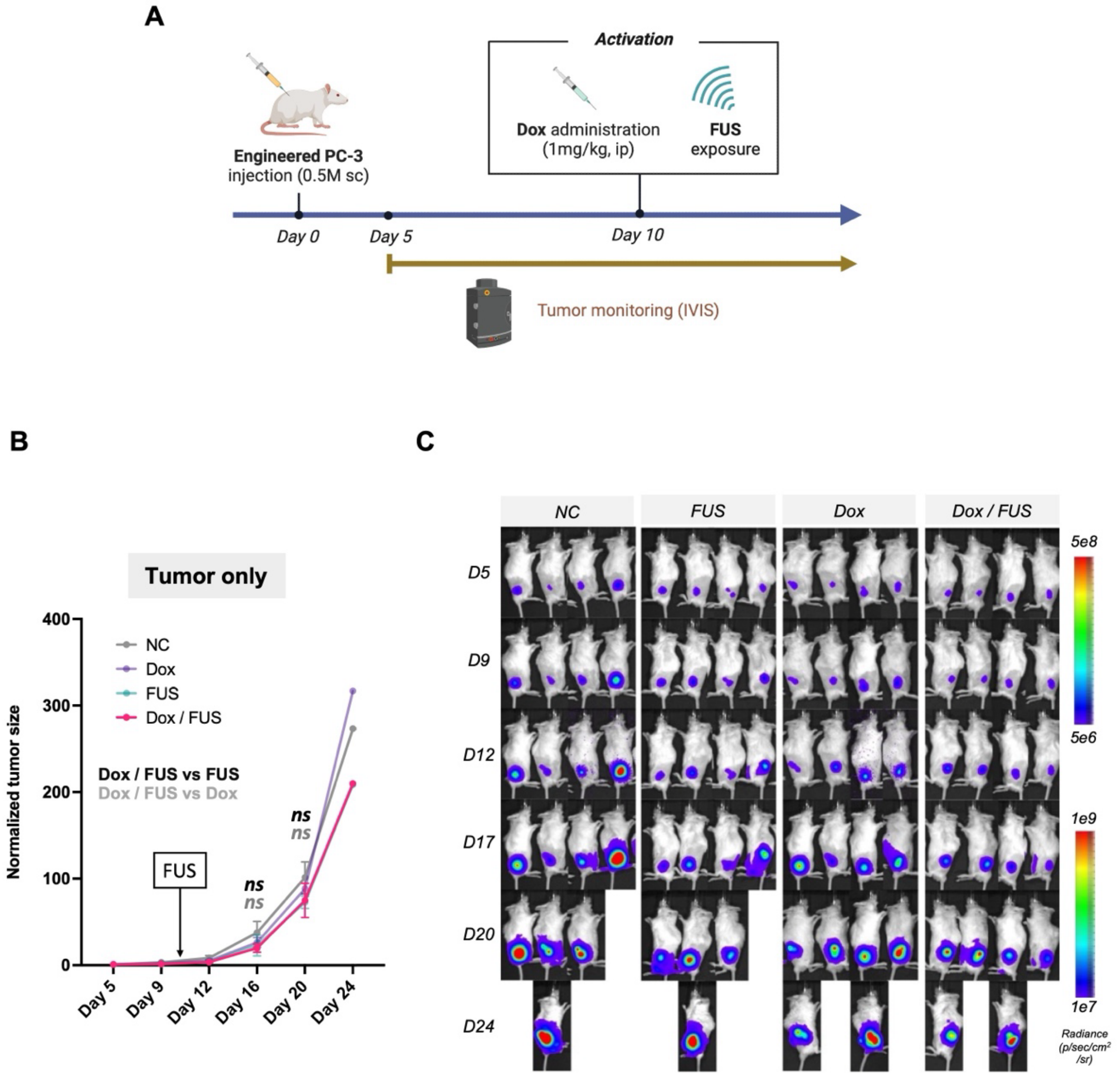

Supplementary Figure 16: Effect of various treatments on tumor growths without synNotch T cell administration. **(A)** Schematics of the experimental timeline. Bilateral tumors were established in NSG mice by subcutaneously injecting PC-3 cells (0.5M per site). Tumors were activated on Day 10 and tumor growth was monitored every three to four days starting from Day 5 using IVIS imaging. **(B)** Normalized tumor size over time, showing the effects of different treatments: no treatment (NC), doxycycline only (Dox), FUS only, and combined doxycycline and FUS treatment (Dox/FUS) (n=4). Data points represent the normalized tumor size at each time point. Error bars represent SEM. Statistical significance was determined by two-way ANOVA with Tukey's multiple comparison test. No significant differences were observed. **(C)** Bioluminescence (BLI) images showing tumor growth in the bilateral tumor model on mice. Created with BioRender.com. Licensed to Yoon, C. (2025) https://BioRender.com/k64u782.

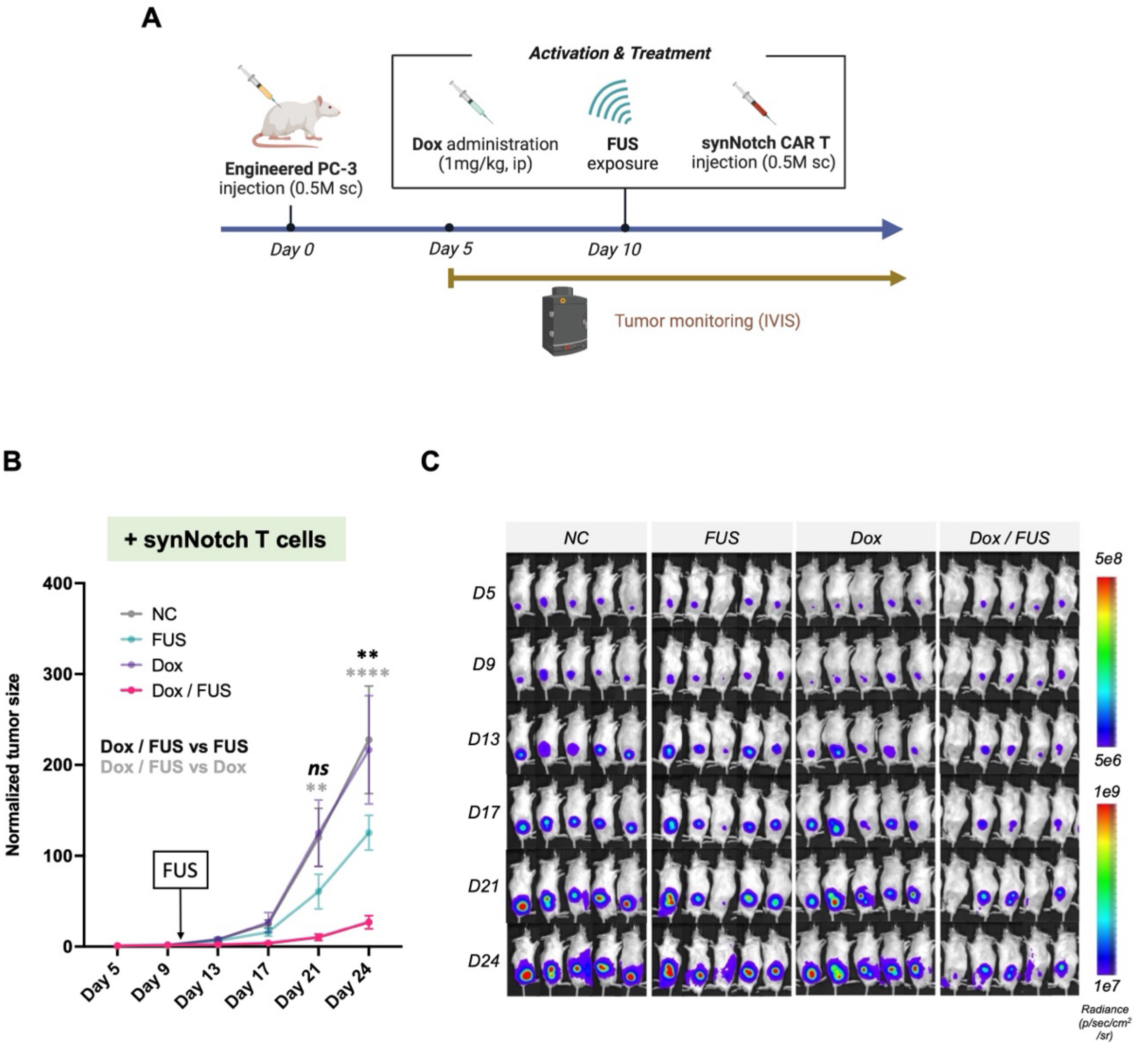

Supplementary Figure 17: Effect of various treatments on tumor growths with synNotch CAR T cell administration. (A) Schematics of the experimental timeline. In addition to the tumor activation on Day 10, synNotch CAR T cells were injected subcutaneously. **(B)** Normalized tumor size over time, showing the effects of different treatments with synNotch CAR T cells: no treatment (NC), FUS only, doxycycline only (Dox), and combined doxycycline and FUS treatment (Dox/FUS) (n=5). Data points represent the normalized tumor size at each time point. Error bars represent SEM. Statistical significance was determined by two-way ANOVA with Tukey's multiple comparison test. At Day 21, Dox/FUS vs. Dox: $p = 0.0010$. At Day 24, Dox/FUS vs. FUS: $p = 0.0062$, Dox/FUS vs. Dox: $p < 0.0001$. **(C)** Bioluminescence (BLI) images showing tumor growth in the bilateral tumor model on mice. Created with BioRender.com. Licensed to Yoon, C. (2025) https://BioRender.com/k64u782.

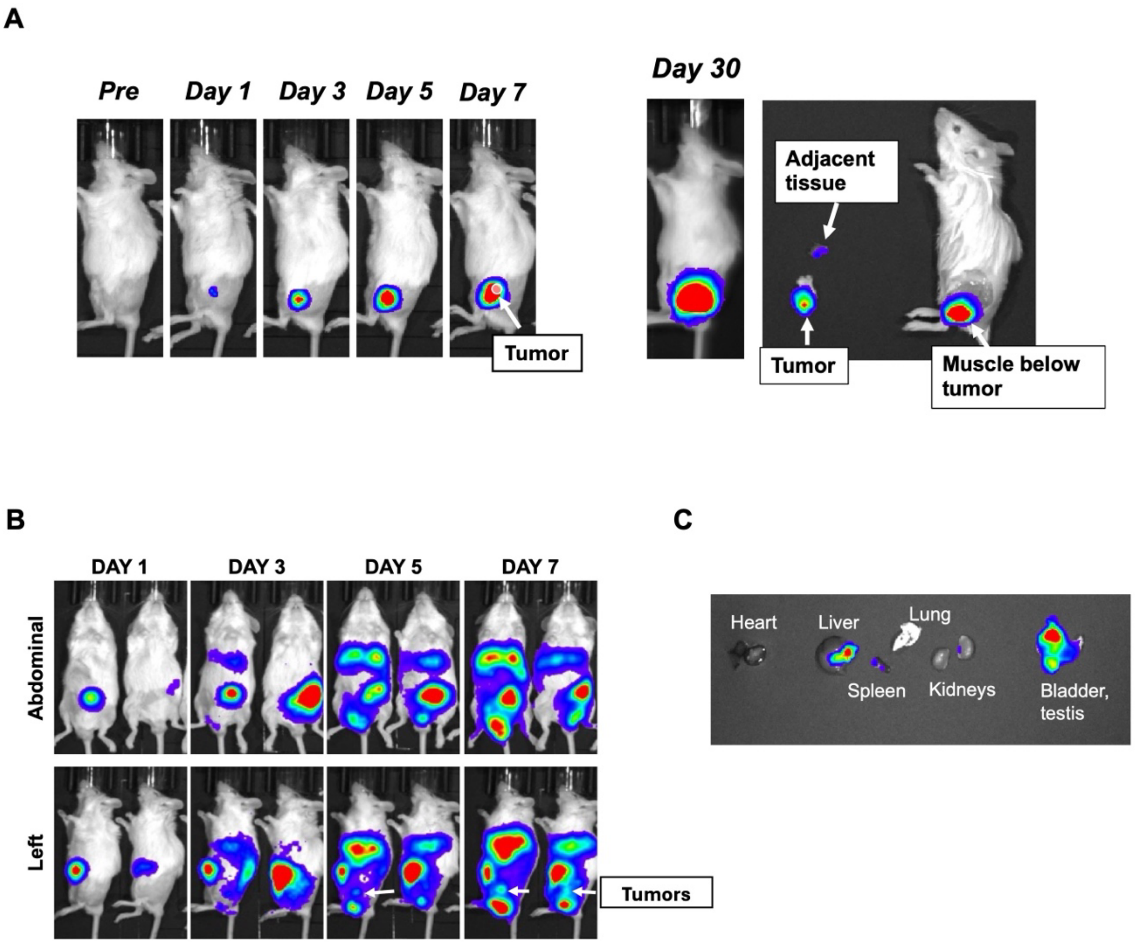

Supplementary Figure 18: Systemic versus focal delivery and distribution of AAV2-CMV-Fluc. Mice were injected with AAV2-CMV-Fluc to assess the delivery and distribution of the viral vector via two different routes: systemic intraperitoneal (i.p.) injection and focal injection. **(A)** In vivo BLI of mice at various time points (Pre, Days 1, 3, 5, 7, and 30) following focal injection of AAV2-CMV-Fluc (2E10 GC per injection, 20 µL, peritumor). The images show initial viral expression localized to the tumor site but progressively spreading beyond the injection site. *Ex vivo* BLI on Day 30 reveals significant leakage of the virus into the muscle below the tumor, indicating that focal injection does not restrict viral distribution strictly to the tumor. **(B)** *In vivo* bioluminescence imaging (BLI) of mice at various time points (Days 1, 3, 5, and 7) following i.p. injection of AAV2-CMV-Fluc (4E11 GC per injection, 100 µL). The images show viral distribution from abdominal and left side views, demonstrating widespread, non-specific infection across multiple body regions. **(C)** *Ex vivo* BLI of dissected organs 30 days post-i.p. injection, confirming viral presence in multiple organs, including the heart, liver, lung, spleen, kidneys, bladder, and testis.

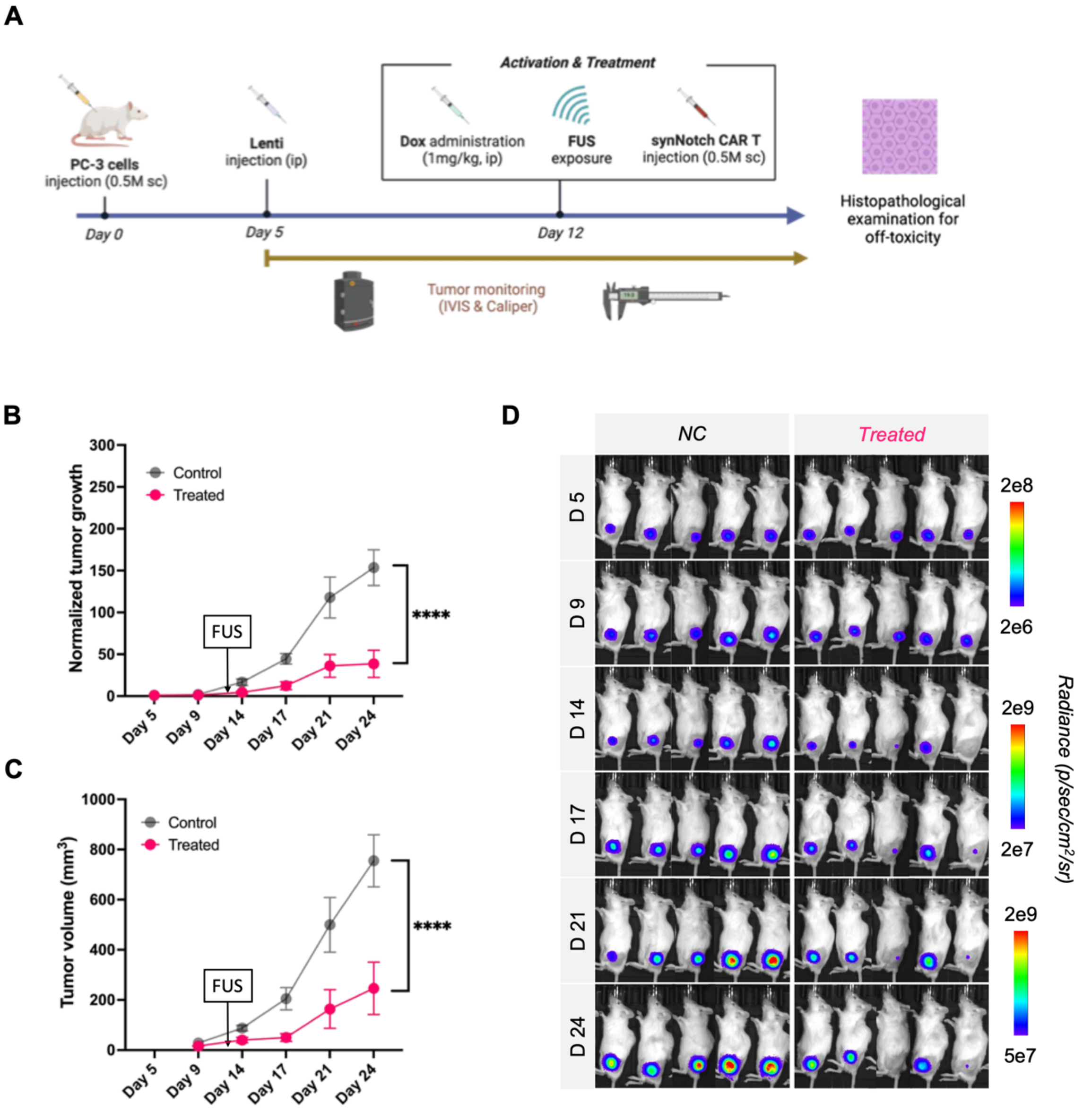

Supplementary Figure 19: ***In vivo*** **evaluation of systemic CaDox delivery with synNotch CAR T cell therapy. (A)** Schematic of the experimental timeline: PC-3 cells were injected subcutaneously into mice, followed by systemic administration of a lentiviral vector encoding the CaDox circuit. Doxycycline was administered, and tumors were exposed to focused ultrasound (FUS) before injecting synNotch CAR T cells. **(B)** Quantification of normalized tumor growth in treated and control groups. **(C)** Tumor volume measurements by caliper at various time points in treated and control groups. **(D)** *In vivo* bioluminescence imaging of tumors at different time points (Days 5, 9, 14, 17, 21, 24) post-tumor inoculation, comparing control and treated groups. Error bars represent SEM. n=5. Statistical significance was determined by two-way ANOVA with Tukey's multiple comparison test. **** indicates adjusted P < 0.0001. Created with BioRender.com. Licensed to Yoon, C. (2025) https://BioRender.com/k64u782.

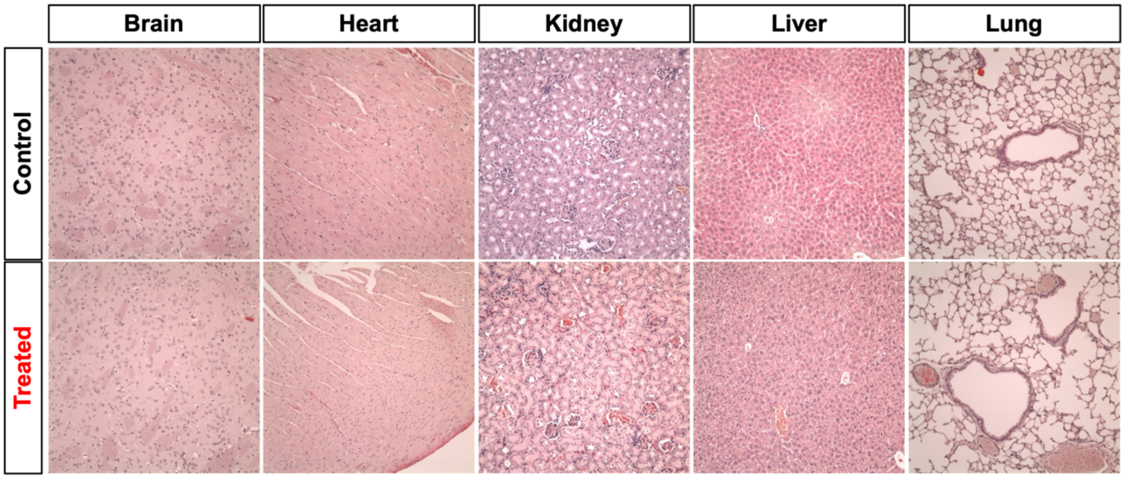

Supplementary Figure 20: Histopathological examination of major organs post-CAR-T Cell infusion to assess off-tumor toxicity. Histopathological analysis of major organs harvested from mice treated with synNotch CAR-T cells. Hematoxylin and eosin (H&E) staining was performed on sections from the brain, heart, kidney, liver, and lung of treated and control mice. No significant off-tumor toxicity or localized tissue damage was observed in any of the organs examined, indicating the safety and specificity of the CAR-T cell activation strategy. The images are representative of multiple fields examined per organ, per animal.

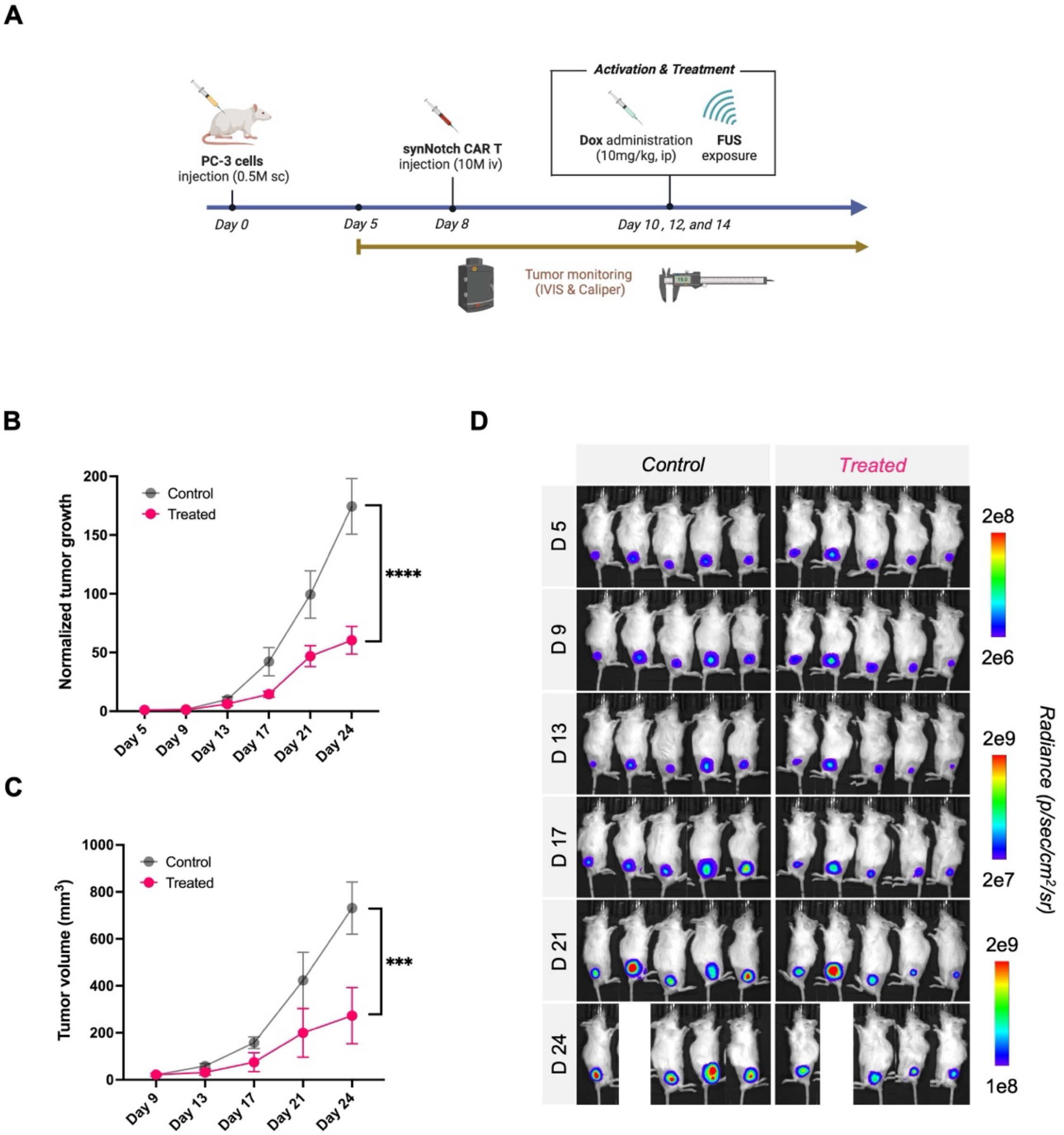

Supplementary Figure 21: ***In vivo*** **evaluation of systemic synNotch CAR T cell delivery and therapeutic effects. (A)** Schematic of the experimental timeline: PC-3 cells were injected subcutaneously into mice, followed by systemic administration (i.v.) of synNotch CAR T cells on Day 8. Doxycycline was administered, and tumors on the left side were exposed to focused ultrasound (FUS) on Day 10, 12, and 14. **(B)** Quantification of normalized tumor growth using IVIS imaging. **** indicates adjusted P < 0.0001 **(C)** Tumor volume measurements using caliper measurement. *** indicates adjusted P = 0.0004. **(D)** In vivo bioluminescence imaging of tumors at different time points (Days 5, 9, 13, 17, 21, 24) post-tumor inoculation, comparing control (Dox-only) and treated (Dox / FUS) groups. Error bars represent SEM. n=5. Statistical significance was determined by two-way ANOVA with Tukey's multiple comparison test. Created with BioRender.com. Licensed to Yoon, C. (2025) https://BioRender.com/k64u782.

Supplementary Tables

Plasmids	Descriptions	Sources
CMV-NFAT(4-460)-EGFP	NFAT translocation tracking	Addgene (#11107)
CMV-NFAT_Variants-EGFP	Kinetic study for NFAT variants	This study
PGK-rtTA-VIVIT_NFAT(4-399)-EGFP	CaDox regulator (EGFP)	This study
TRE-FLuc-PGK-RLuc-mCherry	CaDox reporter (FLuc/RLuc)	This study
TRE-mCherry	CaDox reporter (mCherry)	This study
PGK-rTetR-VIVIT_NFAT(4-399)-VP64-mCherry	CaDox regulator (mCherry)	This study
TRE-NLuc-PGK-EGFP	CaDox reporter (NLuc)	This study
CMV-R-Geco1	Genetically encoded calcium sensor	Addgene (#32444)
TRE-tCD19-EGFP-BSD	CaDox reporter (tCD19)	This study
PGK-αCD19_synNotch-GAL4-VP64	Anti-CD19 synNotch construct	Addgene (#79125)
UAS-BFP-PGK-mCherry	Inducible BFP reporter	Addgene (#79130)
UAS-αPSMA_CAR-PGK-mCherry	Inducible PSMA-CAR reporter	This study

Supplementary Table 1: A list of constructs used in this study.

**Components**	**Final concentration**
50x B27	50x Diluted to make 1x
500 mM N-Acetylcysteine	1.25 mM
0.5 mg/mL EGF	5 ng/mL
100 ug/mL Noggin	100 ng/mL
R-Spondin 1	10% conditioned medium
5 mM A83-01	500 nM
0.1 mg/mL FGF10	10 ng/mL
50 µg/mL FGF2	5 ng/mL
10 mM Prostaglandin E2	1 µM
1M Nicotinamide	10 mM
30 mM SB202190	10 µM
FBS	10%
1000 mM HEPES	10 mM
200 mM GlutaMAX	2 mM
100x Pen-Strep	1x
adDMEM/F12	
100 mM Y-27632 Dihydrochloride	10 µM

Supplementary Table 2: The complete organoid media composition, including all components and their respective concentrations, was adapted from a previously published paper[33] and is presented here.

Supplementary References

1. Broders-Bondon, F., Nguyen Ho-Bouldoires, T.H., Fernandez-Sanchez, M.E. & Farge, E. Mechanotransduction in tumor progression: The dark side of the force. *J Cell Biol* **217**, 1571-1587 (2018).

2. Lv, J. et al. Cell softness regulates tumorigenicity and stemness of cancer cells. *EMBO J* **40**, e106123 (2021).

3. Pfeifer, C.R., Alvey, C.M., Irianto, J. & Discher, D.E. Genome variation across cancers scales with tissue stiffness - an invasion-mutation mechanism and implications for immune cell infiltration. *Curr Opin Syst Biol* **2**, 103-114 (2017).

4. Liu, Q., Luo, Q., Ju, Y. & Song, G. Role of the mechanical microenvironment in cancer development and progression. *Cancer Biol Med* **17**, 282-292 (2020).

5. Moose, D.L. & Henry, M.D. Survival of the resilient: Mechano-adaptation of circulating tumor cells to fluid shear stress. *Mol Cell Oncol* **7**, 1766338 (2020).

6. Chowdhury, F., Huang, B. & Wang, N. Cytoskeletal prestress: The cellular hallmark in mechanobiology and mechanomedicine. *Cytoskeleton (Hoboken)* **78**, 249-276 (2021).

7. Hwang, J.Y. et al. Investigating contactless high frequency ultrasound microbeam stimulation for determination of invasion potential of breast cancer cells. *Biotechnol Bioeng* **110**, 2697-2705 (2013).

8. Weitz, A.C. et al. Functional Assay of Cancer Cell Invasion Potential Based on Mechanotransduction of Focused Ultrasound. *Front Oncol* **7**, 161 (2017).

9. Konofagou, E.E. et al. Ultrasound-induced blood-brain barrier opening. *Curr Pharm Biotechnol* **13**, 1332-1345 (2012).

10. Fan, Z., Liu, H., Mayer, M. & Deng, C.X. Spatiotemporally controlled single cell sonoporation. *Proc Natl Acad Sci U S A* **109**, 16486-16491 (2012).

11. Xu, Z., Hall, T.L., Vlaisavljevich, E. & Lee, F.T., Jr. Histotripsy: the first noninvasive, non-ionizing, non-thermal ablation technique based on ultrasound. *Int J Hyperthermia* **38**, 561-575 (2021).

12. Whitney, N.P., Lamb, A.C., Louw, T.M. & Subramanian, A. Integrin-mediated mechanotransduction pathway of low-intensity continuous ultrasound in human chondrocytes. *Ultrasound Med Biol* **38**, 1734-1743 (2012).

13. Ren, L. et al. Involvement of p38 MAPK pathway in low intensity pulsed ultrasound induced osteogenic differentiation of human periodontal ligament cells. *Ultrasonics* **53**, 686-690 (2013).

14. Louw, T.M., Budhiraja, G., Viljoen, H.J. & Subramanian, A. Mechanotransduction of ultrasound is frequency dependent below the cavitation threshold. *Ultrasound Med Biol* **39**, 1303-1319 (2013).

15. Qiu, Z. et al. The Mechanosensitive Ion Channel Piezo1 Significantly Mediates In Vitro Ultrasonic Stimulation of Neurons. *iScience* **21**, 448-457 (2019).

16. Liao, D., Li, F., Lu, D. & Zhong, P. Activation of Piezo1 mechanosensitive ion channel in HEK293T cells by 30MHz vertically deployed surface acoustic waves. *Biochem Biophys Res Commun* **518**, 541-547 (2019).

17. Ye, J. et al. Ultrasonic Control of Neural Activity through Activation of the Mechanosensitive Channel MscL. *Nano Lett* (2018).

18. Qiu, Z. et al. Targeted Neurostimulation in Mouse Brains with Non-invasive Ultrasound. *Cell Rep* **32**, 108033 (2020).

19. Duque, M. et al. Sonogenetic control of mammalian cells using exogenous Transient Receptor Potential A1 channels. *Nat Commun* **13**, 600 (2022).

20. Locovei, S., Wang, J. & Dahl, G. Activation of pannexin 1 channels by ATP through P2Y receptors and by cytoplasmic calcium. *FEBS Lett* **580**, 239-244 (2006).

21. Locovei, S., Bao, L. & Dahl, G. Pannexin 1 in erythrocytes: function without a gap. *Proc Natl Acad Sci U S A* **103**, 7655-7659 (2006).

22. Furlow, P.W. et al. Mechanosensitive pannexin-1 channels mediate microvascular metastatic cell survival. *Nat Cell Biol* **17**, 943-952 (2015).

23. Agrawal, A. et al. Mechanical signatures in cancer metastasis. *NPJ Biol Phys Mech* **2**, 3 (2025).

24. Bera, K., Kiepas, A., Zhang, Y., Sun, S.X. & Konstantopoulos, K. The interplay between physical cues and mechanosensitive ion channels in cancer metastasis. *Front Cell Dev Biol* **10**, 954099 (2022).

25. Karska, J., Kowalski, S., Saczko, J., Moisescu, M.G. & Kulbacka, J. Mechanosensitive Ion Channels and Their Role in Cancer Cells. *Membranes (Basel)* **13** (2023).

26. Zarogoulidis, P. et al. Suicide Gene Therapy for Cancer - Current Strategies. *J Genet Syndr Gene Ther* **4** (2013).

27. Roybal, K.T. et al. Precision Tumor Recognition by T Cells With Combinatorial Antigen-Sensing Circuits. *Cell* **164**, 770-779 (2016).

28. Wu, Y. et al. Control of the activity of CAR-T cells within tumours via focused ultrasound. *Nat Biomed Eng* **5**, 1336-1347 (2021).

29. Amini, L. et al. Preparing for CAR T cell therapy: patient selection, bridging therapies and lymphodepletion. *Nat Rev Clin Oncol* **19**, 342-355 (2022).

30. Majzner, R.G. et al. GD2-CAR T cell therapy for H3K27M-mutated diffuse midline gliomas. *Nature* **603**, 934-941 (2022).

31. Maude, S.L. et al. Chimeric antigen receptor T cells for sustained remissions in leukemia. *N Engl J Med* **371**, 1507-1517 (2014).

32. Gardner, R.A. et al. Intent-to-treat leukemia remission by CD19 CAR T cells of defined formulation and dose in children and young adults. *Blood* **129**, 3322-3331 (2017).

33. Drost, J. et al. Organoid culture systems for prostate epithelial and cancer tissue. *Nat Protoc* **11**, 347-358 (2016).
