# Supplementary material for: Tumour priming by ultrasound mechanogenetics for CAR T therapy
Source: Nat Mater. 2025 Oct 31;25(2):310–21. doi: 10.1038/s41563-025-02391-8 (PMC12867766; doi:10.1038/s41563-025-02391-8)
Supplement: Supplementary file 2 — Reporting Summary [file 41563_2025_2391_MOESM2_ESM.pdf]

nature portfolio

Corresponding author(s): Yingxiao Wang, Chi Woo Yoon, Longwei Liu

Last updated by author(s): 6/18/2025

Reporting Summary

Nature Portfolio wishes to improve the reproducibility of the work that we publish. This form provides structure for consistency and transparency in reporting. For further information on Nature Portfolio policies, see our Editorial Policies and the Editorial Policy Checklist.

Statistics

For all statistical analyses, confirm that the following items are present in the figure legend, table legend, main text, or Methods section.

n/a	Confirmed	
☐	☒	The exact sample size (*n*) for each experimental group/condition, given as a discrete number and unit of measurement
☐	☒	A statement on whether measurements were taken from distinct samples or whether the same sample was measured repeatedly
☐	☒	The statistical test(s) used AND whether they are one- or two-sided<br>*Only common tests should be described solely by name; describe more complex techniques in the Methods section.*
☒	☐	A description of all covariates tested
☒	☐	A description of any assumptions or corrections, such as tests of normality and adjustment for multiple comparisons
☐	☒	A full description of the statistical parameters including central tendency (e.g. means) or other basic estimates (e.g. regression coefficient) AND variation (e.g. standard deviation) or associated estimates of uncertainty (e.g. confidence intervals)
☐	☒	For null hypothesis testing, the test statistic (e.g. *F*, *t*, *r*) with confidence intervals, effect sizes, degrees of freedom and *P* value noted<br>*Give P values as exact values whenever suitable.*
☒	☐	For Bayesian analysis, information on the choice of priors and Markov chain Monte Carlo settings
☒	☐	For hierarchical and complex designs, identification of the appropriate level for tests and full reporting of outcomes
☒	☐	Estimates of effect sizes (e.g. Cohen's *d*, Pearson's *r*), indicating how they were calculated

Our web collection on statistics for biologists contains articles on many of the points above.

Software and code

Policy information about availability of computer code

Data collection	MetaMorph 7.8 software (Molecular Devices) was used to collect live-cell fluorescence imaging data. Living Image 4.8.2 software (Revvity) was used for acquiring and analyzing in vivo bioluminescence imaging data. COMSOL Multiphysics 6.0 (COMSOL Inc.) was used for acoustic wave simulations, as described in the Methods section.
Data analysis	GraphPad Prism 10.0.0 (GraphPad Software) was used for data graphing and statistical analyses. Flow cytometry data were analyzed and visualized using FlowJo 10.8.2 (BD Biosciences). Imaging data were further analyzed using ImageJ (NIH), CellProfiler 4.2.6 (Broad Institute), and custom scripts written in MATLAB R2023b (MathWorks) and Python 3.12.1. Custom code was used for Cell Response Index (CRI) calculations, mCherry intensity quantification, and ultrasound control. The specific statistical tests and analytical pipelines are described in the Methods and figure legends.

For manuscripts utilizing custom algorithms or software that are central to the research but not yet described in published literature, software must be made available to editors and reviewers. We strongly encourage code deposition in a community repository (e.g. GitHub). See the Nature Portfolio guidelines for submitting code & software for further information.

Data

Policy information about availability of data

All manuscripts must include a data availability statement. This statement should provide the following information, where applicable:

- Accession codes, unique identifiers, or web links for publicly available datasets
- A description of any restrictions on data availability
- For clinical datasets or third party data, please ensure that the statement adheres to our policy

All data generated or analyzed during this study are provided as Source Data or included in the Supplementary information. The Source Data contains the underlying data for all figures in the main text and the Supplementary Information.

Research involving human participants, their data, or biological material

Policy information about studies with human participants or human data. See also policy information about sex, gender (identity/presentation), and sexual orientation and race, ethnicity and racism.

Reporting on sex and gender	Not applicable.
Reporting on race, ethnicity, or other socially relevant groupings	Not applicable.
Population characteristics	Not applicable.
Recruitment	Not applicable.
Ethics oversight	Not applicable.

Note that full information on the approval of the study protocol must also be provided in the manuscript.

Field-specific reporting

Please select the one below that is the best fit for your research. If you are not sure, read the appropriate sections before making your selection.

☒ Life sciences ☐ Behavioural & social sciences ☐ Ecological, evolutionary & environmental sciences

For a reference copy of the document with all sections, see nature.com/documents/nr-reporting-summary-flat.pdf

Life sciences study design

All studies must disclose on these points even when the disclosure is negative.

Sample size	Each experiment was performed with at least three biological replicates. Technical replicates were included where appropriate (e.g., for luciferase and imaging based gene induction assays) to ensure consistency in quantitative measurements. Sample sizes for in vivo experiments were based on prior studies from our group and others using similar xenograft models and therapeutic strategies, which demonstrated that group sizes of 4–6 mice are sufficient to observe biologically meaningful differences (e.g., Wu et al, Nat Biomed Eng BME, 2021; Roybal et al., Cell, 2016; Zhang et al., Nat Biomed Eng, 2015)
Data exclusions	No data was excluded.
Replication	All the experiments has been replicated at least more than three to ensure the reproducibility.
Randomization	Randomization was not applied to in vitro experiments, as they were conducted under controlled and uniform conditions. For in vivo studies, allocation to experimental groups was randomized based on baseline tumor size. Mice with comparable tumor volumes were evenly distributed across treatment groups to ensure balance. Additionally, animals from different cages were included in each group to minimize cage-specific environmental effects.
Blinding	Investigators were not blinded to group allocation during data collection and analysis because key outcome measures (e.g., tumor size, fluorescence intensity) were objectively quantifiable using standardized imaging and analysis tools. Given the focus on feasibility and mechanistic validation, the potential for subjective bias was minimized through consistent protocols.

Reporting for specific materials, systems and methods

We require information from authors about some types of materials, experimental systems and methods used in many studies. Here, indicate whether each material, system or method listed is relevant to your study. If you are not sure if a list item applies to your research, read the appropriate section before selecting a response.

Materials & experimental systems

n/a	Involved in the study
☐	☒ Antibodies
☐	☒ Eukaryotic cell lines
☒	☐ Palaeontology and archaeology
☐	☒ Animals and other organisms
☒	☐ Clinical data
☒	☐ Dual use research of concern
☒	☐ Plants

Methods

n/a	Involved in the study
☒	☐ ChIP-seq
☐	☒ Flow cytometry
☒	☐ MRI-based neuroimaging

Antibodies

Antibodies used

anti-CD19 (clone HIB19) monoclonal antibody conjugated with APC (BioLegend, 302218, 1:20), anti-CD19 (clone HIB19) monoclonal antibody conjugated with AF647 (BioLegend, 302220, 1:100), anti-c-Myc (clone 9E10) antibody conjugated with AF647 (BioLegend, 626809, 1:20)

Validation

All primary antibodies used in this study are commercially available and have been validated by the manufacturers for use in human samples and the relevant applications. The anti-CD19 monoclonal antibody (clone HIB19, APC-conjugated; BioLegend, 302218) is validated for flow cytometry and has been cited in multiple peer-reviewed studies, including Chabi et al., Cell Reports, 2020; 29(8):2307–2320 and Sefik et al., Nature, 2022; 606:585. The anti-CD19 monoclonal antibody (clone HIB19, AF647-conjugated; BioLegend, 302220) is validated for both immunofluorescence and flow cytometry, with validation supported by Segaliny et al., Commun Biol., 2023; 6:380 and Nerreter et al., Nat Commun., 2019; 10:3137. The anti-c-Myc monoclonal antibody (clone 9E10, AF647-conjugated; BioLegend, 626809) is validated for flow cytometry and has been cited in Merljak et al., Nat Commun., 2023; 14:1995 and Gioulbasani et al., Commun Biol., 2024; 1606:7. In addition to these manufacturer-provided validations, antibody performance was confirmed in-house through consistent detection of inducible targets following stimulation, with expected expression patterns.

Eukaryotic cell lines

Policy information about cell lines and Sex and Gender in Research

Cell line source(s)

The cell lines used in this study, including HEK293T (CRL-3216), PC-3(CRL-1435), Jurkat T(TIB-152), U-87MG (HTB-14), and MDA-MB-231 (CRM-HTB-26) cells, were obtained from the American Type Culture Collection (ATCC). Lenti-X 293T (632180) cells were acquired from the Clontech Laboratories.

Authentication

The cell lines used in this study, including HEK293T (CRL-3216), PC-3 (CRL-1435), Jurkat T (TIB-152), U-87MG (HTB-14), and MDA-MB-231 (CRM-HTB-26), were obtained from the American Type Culture Collection (ATCC), which performs authentication using short tandem repeat (STR) profiling and morphology validation. The Lenti-X 293T cell line (632180) was purchased from Clontech Laboratories, which also provides authenticated cells. No additional in-house STR profiling or DNA barcoding was conducted, as we relied on authenticated, verified sources and used the cells within early passages after receipt.

Mycoplasma contamination

All cell lines were tested negative for mycoplasma contamination.

Commonly misidentified lines
(See ICLAC register)

No commonly misidentified cell lines used in this study.

Animals and other research organisms

Policy information about studies involving animals; ARRIVE guidelines recommended for reporting animal research, and Sex and Gender in Research

Laboratory animals

NOD scid gamma (NSG) mice (6–8 weeks old, male), obtained from UCSD Animal Care Program (ACP) or the Jackson Laboratory, were utilized for all animal experiments. Mice were housed under specific pathogen-free conditions in individually ventilated cages, with a 12-hour light/12-hour dark cycle, ambient temperature of 20–24□°C, and relative humidity of 40–60%.

Wild animals

No wild animals were used in this study.

Reporting on sex

Male only.

Field-collected samples

No field-collected samples were used in this study.

Ethics oversight

Animal experiments were conducted in accordance with either Protocol S15285, approved by the UCSD Institutional Animal Care and Use Committee (IACUC) or Protocol 21479, approved by the USC IACUC.

Note that full information on the approval of the study protocol must also be provided in the manuscript.

Plants

Seed stocks	Not applicable.
Novel plant genotypes	Not applicable.
Authentication	Not applicable.

Flow Cytometry

Plots

Confirm that:

☒ The axis labels state the marker and fluorochrome used (e.g. CD4-FITC).

☒ The axis scales are clearly visible. Include numbers along axes only for bottom left plot of group (a 'group' is an analysis of identical markers).

☒ All plots are contour plots with outliers or pseudocolor plots.

☒ A numerical value for number of cells or percentage (with statistics) is provided.

Methodology

Sample preparation	Samples from different groups were washed with DPBS, incubated with antibodies, if needed, in 4 degree for 30 minutes before further washing with DPBS three times.
Instrument	BD Accuri C6 Plus flow analyzer
Software	FlowJo Software (FlowJo LLC)
Cell population abundance	At lease 20,000 events of singlets.
Gating strategy	Dead cells, and doublets were excluded before analysis. Appropriate controls were utilized to separate positive and negative populations.

☒ Tick this box to confirm that a figure exemplifying the gating strategy is provided in the Supplementary Information.
